# Noninvasive western lowland gorilla's health monitoring: A decade of simian immunodeficiency virus surveillance in southern Cameroon

**DOI:** 10.1002/ece3.4478

**Published:** 2018-10-25

**Authors:** Christian Julian Villabona‐Arenas, Ahidjo Ayouba, Amandine Esteban, Mirela D'arc, Eitel Mpoudi Ngole, Martine Peeters

**Affiliations:** ^1^ TransVIHMI Institut de Recherche pour le Développement (IRD) Institut national de la santé et de la recherche médicale (INSERM) Université de Montpellier Montpellier France; ^2^ Centre de recherche sur les maladies émergentes et réémergentes (CREMER) Institut de Recherches Médicales et d'Etudes des Plantes Médicinales (IMPM) Yaoundé Cameroun

**Keywords:** Cameroon, microsatellites, noninvasive sampling, phylogeny, SIVgor, western lowland gorillas

## Abstract

Simian immunodeficiency virus (SIVgor) causes persistent infection in critically endangered western lowland gorillas (*Gorilla gorilla gorilla*) from west central Africa. SIVgor is closely related to chimpanzee and human immunodeficiency viruses (SIVcpz and HIV‐1, respectively). We established a noninvasive method that does not interfere with gorillas' natural behaviour to provide wildlife pathogen surveillance and health monitoring for conservation. A total of 1,665 geo‐referenced fecal samples were collected at regular intervals from February 2006 to December 2014 (123 sampling days) in the Campo‐Ma'an National Park (southwest Cameroon). Host genotyping was performed using microsatellite markers, SIVgor infection was identified by serology and genetic amplification was attempted on seropositive individuals. We identified at least 125 distinct gorillas, 50 were resampled (observed 3.5 times in average) and 38 were SIVgor+ (seven individuals were seroconverters). Six groups of gorillas were identified based on the overlapping occurrence of individuals with apparent high rates of gene flow. We obtained SIVgor genetic sequences from 25 of 38 seropositive genotyped gorillas and showed that the virus follows exponential growth dynamics under a strict molecular clock. Different groups shared SIVgor lineages demonstrating intergroup viral spread and recapture of positive individuals illustrated intra‐host viral evolution. Relatedness and relationship genetic analysis of gorillas together with Bayesian phylogenetic inference of SIVgor provided evidence suggestive of vertical transmission. In conclusion, we provided insights into gorilla social dynamics and SIVgor evolution and emphasized the utility of noninvasive sampling to study wildlife health populations. These findings contribute to prospective planning for better monitoring and conservation.

## 1 INTRODUCTION

Simian immunodeficiency viruses (SIVs) have been isolated from many wild African non‐human primates species, each infected with a species‐specific SIV (Locatelli & Peeters, 2012; Sharp & Hahn, 2011). The closest simian relatives of HIV‐1 are SIVcpz from chimpanzees and SIVgor from gorillas, which each have been transmitted to humans in at least two occasions: SIVcpz strains from central chimpanzees (*Pan troglodytes troglodytes*) in southern Cameroon are the ancestors of the pandemic HIV‐1 group M and the geographically restricted group N, whereas SIVgor strains from gorillas in Cameroon are the ancestors of HIV‐1 group O and P, which each are also restricted to that country (D'Arc et al., 2015; Keele et al., 2006; Sharp & Hahn, 2011; Van Heuverswyn et al., 2006, 2007). In turn, SIVgor originated after a cross‐species transmission of SIVcpz (D'Arc et al., 2015; Takehisa et al., 2009). Infection with SIVcpz is common in central and eastern chimpanzees (*P.t. schweinfurthii*) that are distributed in central Africa countries, but SIVgor is restricted to critically endangered western lowland gorillas (*Gorilla gorilla gorilla*) from southern Cameroon (D'Arc et al., 2015; Keele et al., 2006; Li et al., 2012; Neel et al., 2010; Santiago et al., 2003; Van Heuverswyn et al., 2007). It is unclear whether the current distribution of SIVgor is related to a recent cross‐species event, geographic barriers or host population decline due to pathogenicity. Previous studies on habituated wild eastern chimpanzees in Tanzania showed that SIVcpz infection has a negative impact on the health, reproduction, and survival of chimpanzees and caused the decline of one community (Keele et al., 2009; Rudicell et al., 2010; Terio et al., 2011). AIDS‐related symptoms have also been observed in a naturally infected central chimpanzee from Cameroon (Etienne et al., 2011). Studies on the impact of SIVgor infection are scarce but they are much needed in order to understand gorilla population dynamics.

The integration of research on infectious diseases and primate ecology provides not only benefits for gorillas' conservation but also provides insights into the role of disease in primate evolution and into the emergence of disease in humans (Leendertz et al., 2006). A major issue to monitor health in western lowland gorillas is the lack of habituated SIVgor infected populations. Nonetheless, there is the possibility of acquiring indirect information related to SIVgor infection on a cross‐sectional basis using noninvasive sampling. During previous surveys conducted between 2004 and 2011, we identified several SIVgor infected gorillas in the Campo Ma'an National Park in southwest Cameroon (Neel et al., 2010). We demonstrated the feasibility of long‐term monitoring of SIVgor infection in nonhabituated gorillas and we showed for the first time that it is possible to re‐sample gorillas in a noninvasive way and to characterize SIVgor diversity (Etienne et al., 2012). Here we included additional 4 years of follow‐up, inferred groups of gorillas using geo‐referenced data over time and performed genetic and phylogenetic analyses using state‐of‐the‐art approaches. We sought to advance our understanding of SIVgor transmission and evolution among gorillas in the Campo‐Ma'an National Park and to further validate a noninvasive approach to better monitor and inform the conservation of this critically endangered and elusive species.

## 2 METHODS

### 2.1 Study site and sample collection

The Campo‐Ma'an National Park is located in the South Western corner of Cameroon, bordering on Equatorial Guinea to the south and the Atlantic Ocean to the west covering an area of approximately 2,640 km^2^. This study was conducted in an area of approximately 200 km^2^ at the center of the park where we previously characterized SIVgor infected gorillas (Etienne et al., 2012). Opportunistic collection of fecal samples occurred between 07:00 and 17:00 hr. Field data included sample's GPS coordinates, location type (nest, feeding site or directly on track), estimated time of fecal deposition and, since 2012, the hour of collection. About 20 g of fecal material was collected in a 50‐ml tube, containing 20 mL of RNA*later* ^™^ (Ambion, Austin, TX, USA), and kept at ambient temperature, for a maximum of 2 weeks, and then stored frozen.

### 2.2 Detection of SIVgor antibodies and viral RNA in fecal samples

The fecal samples were tested with the INNO‐LIA^™^ HIV I/II score (Innogenetics, Ghent, Belgium) to survey the presence of cross‐reactive antibodies against HIV‐1. Several independent RNA extractions were performed for each specimen with reactive antibodies, followed by multiple RT‐PCR attempts to increase the chance of SIVgor genetic amplification. Total nucleic acids were extracted with the NucliSens system (Biomérieux, Craponne, France), and RT‐PCR reactions were performed using SIVgor and SIVgor/SIVcpz/HIV‐1 consensus primers that targeted the *env* (200, 315 or 440 bp of the gp41 transmembrane envelope protein's ectodomain) and *pol* (245 or 330 bp of the integrase) regions as previously described (D'Arc et al., 2015; Etienne et al., 2012; Neel et al., 2010). cDNA were generated with Expand reverse transcriptase and Expand long‐template PCR system (Roche Diagnostics, Indianapolis, IN, USA) according to the manufacturer's instructions. For each RT reaction, we used 10 μl of fecal viral RNA extract that we first incubated with 40 pmol of the outer reverse R1 primer for 10 min at 65°C, rapidly cooled on ice. We then added this mixture to the RT‐PCR components, including 20 U of RNase inhibitor (Applied Biosystems/Ambion), in a final volume of 20 μl. The mix was finally incubated for 60 min at 42°C, followed by 5 min at 95°C to inactivate the enzyme. Ten (10) μl of the reverse‐transcribed RNA was used for downstream first round PCR using F1 and R1 primers. For the second round of the nested PCR, we used 5 μl of the first round PCR product in a final reaction volume of 50 μl. PCR amplifications included 45 cycles of denaturation (94°C, 20 s), annealing (45–55°C, depending on the primer Tm for 30 s), and elongation (68°C, 1 min) in a thermal cycler. PCR products were purified and directly sequenced using an automated sequencer (3,130 × l or 3,150 Genetic Analyser; Applied Biosystems, Foster City, CA, USA).

### 2.3 Genetic characterization of Gorillas

Sex determination was performed using PCR products generated from the *amelogenin* gene that contains a deletion in the X, but not in the Y chromosome as previously described (Etienne et al., 2012). Host genotyping was performed using seven microsatellite loci (D18S536, D4S243, D10S676, D9S922 D2S1326, D2S1333, and D4S1627). To minimize allelic dropout, three to seven amplications were performed on homozygous loci. When PCR reactions yielded poor results, a new set of PCRs was performed on a new fecal nucleic acids extract. Samples that did not provide any successful result after five PCR attempts and two independent DNA extractions were discarded and considered as degraded. Samples with an incomplete allelic profile (less than four loci) or mixed profile were also discarded from further analyses.

Seven additional microsatellite loci (vWF, D7s817, D7s2204, D16s2624, D8s1106, D10s1432, and D1s550) were obtained from at least one nucleic acids extract of each gorilla to improve the estimation of relatedness and relationship among the different individuals. Genetic diversity was quantified by estimating observed and expected heterozygosis. Test for Hardy–Weinberg equilibrium (HWE) for each locus and test for linkage disequilibrium between loci were performed using the package adegenet (Jombart, 2008; Jombart & Ahmed, 2011).

A Minimum Spanning Network (MSN) of microsatellite haplotypes was constructed using the Prevosti (Prevosti, Ocana, & Alonso, 1975) and the Bruvo's distance (Bruvo, Michiels, D'Souza, & Schulenburg, 2004) for its ability to handle missing data and that were included in the package (Kamvar, Brooks, & Grunwald, 2015; Kamvar, Tabima, & Grunwald, 2014). The relatedness value (*r*_d_) was calculated using the Wang estimator (Wang, 2017; Wang, 2002) included in the package Demerelate (Kraemer & Gerlach, 2017), the Maximum Likelihood (ML) approach implemented in the ML‐relate software (Kalinowski, Wagner, & Taper, 2006), and Bayesian estimation using the package SOLOMON (Christie, Tennessen, & Blouin, 2013). The Wang estimator was chosen because it is robust for small samples sizes and applies to any number of alleles per locus and to any allele frequency distribution (J. Wang, 2017; J. L. Wang, 2002). The Bayesian parentage analysis requires the prespecification of adults and offspring genotypes. To account for this, we randomly distributed our data in two groups and then performed the analysis. We repeated this approach ten times and preferred the pairs that were hypothesized as parent‐offspring in any of them; a cutoff value of 0.05 was chosen for the posterior probability of a pair being false given frequencies of shared alleles.

### 2.4 Clustering of individuals to reconstruct gorilla groups

At every sampling day, the sample's geographical coordinates were used to trace individuals and to cluster them. We used distance values ranging from 10 to 500 m. We used the estimated time of fecal deposition to more accurately trace the occurrence of gorillas over time (e.g., an estimated fecal deposition of 24 hr changed an individual's occurrence to the preceding day). The clustering of gorillas was performed following a stepwise procedure coded in *R* (R Core Team, 2014). Gorillas were assigned to the same group when their traced ranges overlapped at a given time‐point. Each time an individual with a membership was recaptured, any other individual observed together was added to the corresponding group. The outcomes of the clustering algorithm were branching diagrams for every sampling day (which were generated using the geodesic distance and distance‐matrix methods) and the group's identity of each gorilla using the prespecified distance cutoff. We further analyzed the demarcation of groups over time and scrutinized those cases where two groups merged by examining the corresponding field notes and excluding observations in ensuing sensitivity analysis.

### 2.5 SIV Phylogenetic analysis

The novel and previous SIVgor genetic sequences were used in reconstruction of phylogenetic trees. When multiple sequence reads from the same individual and the same day were available, consensus sequences were generated and used in time‐scaled evolutionary analyses following Markov chain Monte Carlo sampling as implemented in BEAST v1.8.3 software package (Drummond, Suchard, Xie, & Rambaut, 2012). Reconstructions were performed using both genetic regions. Regressions of root‐to‐tip genetic distance against sampling time for each of the datasets were performed using TempEst v1.5.1 (Rambaut, Lam, de Carvalho, & Pybus, 2016) and trees constructed with maximum‐likelihood algorithms in FastTree 2.1 (Price, Dehal, & Arkin, 2009, 2010). All datasets exhibited positive correlation between genetic divergence and sampling time and appeared to be suitable for phylogenetic molecular clock analysis (correlation coefficient of 0.29 and 0.14 for *gp41* and *pol,* respectively). The datasets were analyzed using a *HKY*+*4Γ (*Hasegawa, Kishino, & Yano, 1985 *)* nucleotide substitution model plus a demographic coalescent tree prior model (among the parametric constant size, exponential or logistic growth models and the nonparametric Skyride one) (Drummond, Rambaut, Shapiro, & Pybus, 2005; Minin, Bloomquist, & Suchard, 2008) and either a molecular strict clock model or a relaxed uncorrelated lognormal one (Drummond, Ho, Phillips, & Rambaut, 2006). Model comparisons were performed using the path sampling and stepping stone approach (A minimum of 50 steps with the length of each chain being at least one million iterations) (Baele et al., 2012). For each dataset, two to four Markov chain Monte Carlo chains of 20–200 million iterations were computed. Samples were combined and diagnosed using visual trace inspection and calculation of effective sample sizes (>200) in Tracer (Rambaut, Drummond, Xie, Baele, & Suchard, 2018).

## 3 RESULTS

### 3.1 Individual identification

**Figure 1:**
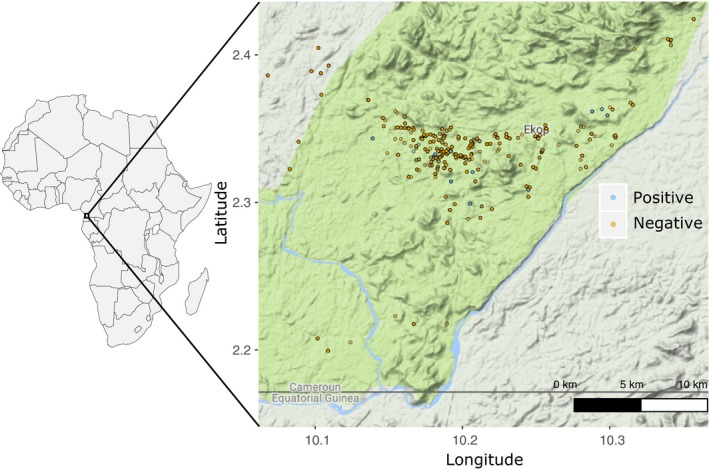
Location of 1,665 wild western lowland gorilla (Gorilla gorilla gorilla) fecal samples collected in the Campo‐Ma'an National Park between February 2006 and December 2014. SIVgor serological findings are indicated in colours

In addition to the initial visit in 2004 where we identified the first SIVgor infected gorilla (CPg‐ID001), a total of 24 visits to the Campo‐Ma'an National Park were conducted between February 2006 and December 2014. There were on average three visits per year (Median 3, IQR 2–3, Maximum = 4) that lasted around five consecutive days (Median 5, IQR 3‐7, Maximum = 8) for a total of 123 sampling days. A total of 1,665 fecal samples were collected in this period with complete geo‐referenced data (Figure 1). A genotype accumulation curve showed that with seven loci we reached a plateau and had enough power to discriminate 125 distinct individuals (Supporting Information Figure S1). A total of 50 gorillas were recaptured and were observed 3.5 times in average (3rd Quartile = 5, Maximum = 9). Table 1 shows that we have in average 8.6 microsatellite alleles per locus. Most loci have <8% missing observations; loci D7s817, D7s2204, and D10s1432 had between 17 and 23 percent missing observations. Overall, loci were highly polymorphic and evenly distributed. Most loci were under the null expectation of Hardy–Weinberg equilibrium (*p *>* *0.05) except for loci vWF, D7s817, D8s1106, D10s1432, and D2S1333 (Table 1). Supporting Information Figure S1 underscores that most individuals were heterozygous for each locus and that loci were not linked (low *r*_d_ value).

**Table 1:** Overview of the genetic parameters for the 14 loci

Locus^a^	Missing data (%)	Number of alleles	Simpson diversity (1‐D)	Nei's 1978 gene diversity	Evenness	Hardy–Weinberg equilibrium (exact *p‐*value)
*D18S536*	0.0	6	0.66	0.67	0.80	0.550
*D4S243*	0.79	8	0.78	0.79	0.84	0.866
*D10S676*	2.38	8	0.72	0.73	0.84	0.532
*D9S922*	4.76	16	0.79	0.79	0.57	0.065
*D2S1326*	9.79	11	0.83	0.83	0.80	0.192
*D2S1333*	3.97	11	0.84	0.84	0.81	0.045
*D4S1627*	6.35	9	0.78	0.78	0.81	0.297
*vWF*	7.94	8	0.74	0.74	0.75	0.015
*D7s817*	22.22	9	0.84	0.85	0.92	0.025
*D7s2204*	17.46	9	0.70	0.70	0.66	0.525
*D16s2624*	3.97	6	0.63	0.63	0.81	0.519
*D8s1106*	7.94	5	0.64	0.64	0.78	0.004
*D10s1432*	18.25	7	0.71	0.72	0.72	0.000
*D1s550*	7.14	7	0.73	0.73	0.75	0.373
Mean	7.43	8.57	0.74	0.75	0.78	…

a Underlined loci were used for host genotyping. The remaining loci were used to improve the estimation of relatedness and relationship among the different individuals.

### 3.2 Reconstruction of gorilla groups over time

**Figure 2:**
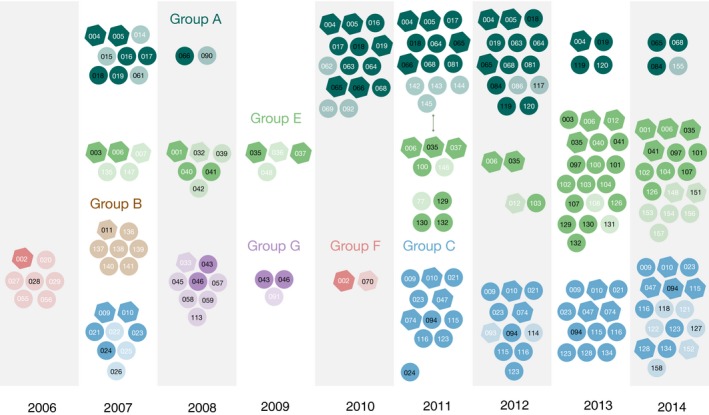
Inferred groups of wild western lowland gorillas in the Campo‐Ma'an National Park based on the overlapping occurrence of individuals. Every colour represents a group. Only the last sampling point of every year is presented for clarity. Hexagons represent individuals with reactive SIVgor antibodies whereas circles represent individuals with non‐reactive ones. White numbers denote females and black numbers denote males. Light fill for individuals sampled once and dark fill for individuals sampled multiples times. The connecting line indicates when group A and group E intersected

Our clustering algorithm grouped 114 gorillas into six groups (Figure 2). The remaining 11 gorillas clustered separately with individuals without successful genotyping. The groups' names were selected to match as closely as possible those from Etienne et al. (Etienne et al., 2012) The smallest group (group B) comprised seven members while the largest group (group E) comprised 37 members. However, this number represents a 9‐year sampling period. The number of individuals varied by sampling point and the highest number of members observed at any single time was 14. Members of Group B were only detected once in 2007 while members of groups F and G were detected in 2006/2010 and 2008/2009, respectively. Member of groups A, C, and E was consistently re‐sampled over more than 5 years. The samples from individual CPg‐001 spanned a decade (2004, 2008, and 2014).

The ranges of Groups A and E intersected once during a sampling day in May 2011 as a cause of sample CR6687 (CPg‐035). Both groups kept their original identity when this sample was removed in sensitivity analyses. Sample CR6687 was found directly on track rather than a nesting site so its occurrence next to samples from another group may reflect a chance event rather than the existence of a unique larger group. The ranges of Groups A and E also overlapped once when using a range ≥200 m due to the proximity of individuals in a single sampling day during February 2007. However, both groups kept their distinctiveness when this observation was removed in sensitivity analysis. It is worth mentioning that western lowland gorillas occasionally from “nesting super‐groups” where two groups nest at distances of 30–50 m (Bermejo, 2004).

Most groups were polygynous. For instance, the most representative samples (i.e., having at least five members) of groups A, B, C, and F had between one or three males and the number of females ranged between four and 11 (Figures 2, Supporting Information Figures S2–S4). Females were still greater in number in the most representative samples of groups E but there was a sample where both sexes were equally represented and two samples with a larger number of males (Supporting Information Figure S5). Group G only representative sample had a disproportionate number of males: one female and seven males were observed at a single time point.

The percentage of individuals with SIVgor infection in the most representative samples ranged between 10% and 60%; in average, 30%of individuals were SIVgor positive at any given point (Figure 2, Supporting Information Figures S2–S5).

### 3.3 Interindividual relatedness

**Figure 3:**
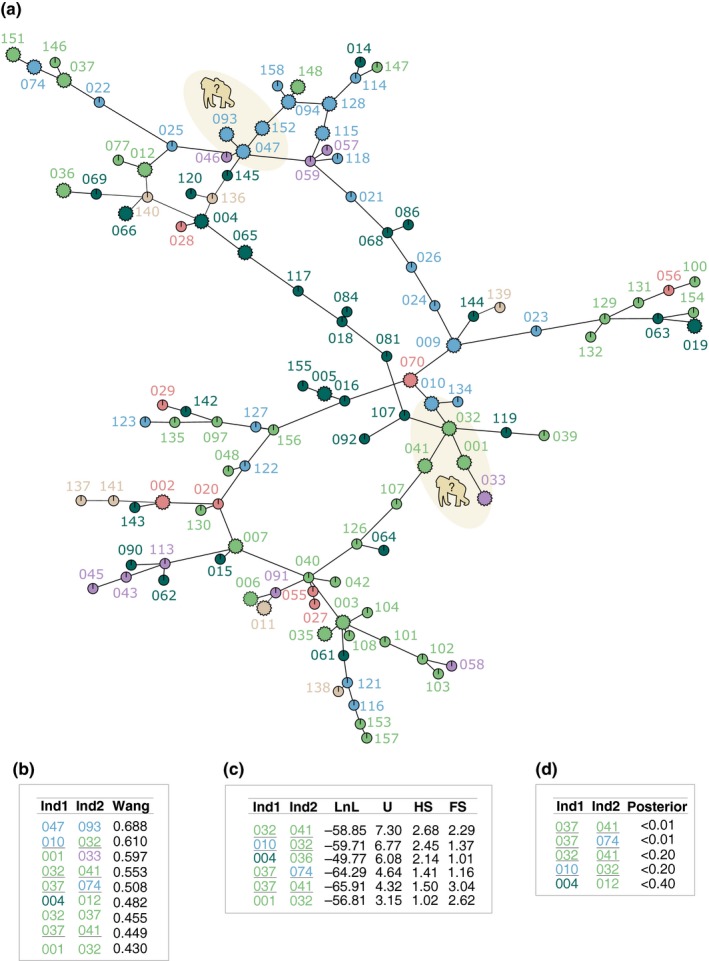
Genetic relatedness of wild western lowland gorillas in the Campo‐Ma'an National Park. (a) minimum spanning network using microsatellite loci and Prevosti's distance. Hexagons represent individuals with reactive SIVgor antibodies whereas circles represent individuals with non‐reactive ones. The gorilla silhouettes indicate instances of apparent vertical transmission. (b) highest pair‐wise relatedness estimated using Wang distance. Parent‐offspring relationships inferred using either Maximum Likelihood (c) or Bayesian methods (d). In the case of the Maximum Likelihood analysis, the probability difference for each individual pair being nonrelated, half‐siblings and full‐siblings is also presented. In the case of the Bayesian analysis. Pairs that were consistently identified by all bottom metrics are underscored. Individuals are coloured according to the groups in Figure 2

A MSN of microsatellites using Prevosti's distance is shown in Figure 3. This MSN shows that genetically close haplotypes did not match the groups that were delineated using the overlapping occurrence of individuals. Similar findings were observed with the MSN of microsatellites using Bruvo's distance (Supporting Information Figure S6).

We used three different metrics to assess interindividual's relatedness and we retained the pairs that had the highest estimates. In the Wang's metric, we kept the pairs with an estimated value over 0.6. In the ML calculations, we kept the pairs that were discriminated as parent–offspring pairs and where the log likelihood of the individuals being unrelated was at least seven times less than the log likelihood of the individuals being parent–offspring. In the Bayesian calculations, a cutoff value of 0.05 was chosen for the posterior probability of a pair being false given frequencies of shared alleles. Under these constrains, there were 23 different pairs of gorillas that had a close genetic relationship by at least one metric and that were relatives in the MSN. Eight pairs were proposed by two methods and four pairs were proposed by all three methods (CPg‐ID010/CPg‐ID134, CPg‐ID019/CPg‐ID063, CPg‐ID019/CPg‐ID154, and CPg‐ID032/CPg‐ID119). The individuals in each of the 23 pairs belonged to the same inferred group in 56% of the cases. Three pairs have both individuals SIVgor positive (CPg‐ID001/CPg‐ID033, CPg‐ID047/CPg‐ID093, and CPg‐ID047/CPg‐ID152 and all were females), and 10 pairs have one individual SIV positive. Among noninfected SIVgor pairs there were six, three and one female/female, female/male, and male/male pairs, respectively. The same proportions were observed among the ten pairs with one individual SIVgor positive.

Supporting Information Figure S2 shows the genetic relatedness of individuals as networks for groups A, B, and E. The percentage of individuals than fell within the largest network in each group (a proxy of the family history) ranged between 21% and 48%, whereas the percentage of individuals in each group that were nonrelated ranged between 25% and 69%. In average, the percentage of individuals in each group that were sampled once was of 47% and among them between 64% and 100% did not fell within the largest network. The percentage of reactive SIVgor individuals from each group that fell within the corresponding largest network ranged between 20% and 78%. The males that consistently have the largest number of connecting edges were CPg‐018, CPg‐094, and CPg‐032 for groups A, C, and E, respectively.

We further dissected the genetic relatedness of individuals over time in order to elucidate any family history. In the case of group A (Supporting Information Figure S3), we consistently sampled females CPg‐004, CPg‐017, CPg‐019, and CPg‐068. These females ended‐up connected to the male CPg‐018 through individuals CPg‐064, CPg‐065, CPg‐081, and CPg‐084, which were sampled later and may represent the offspring. Similarly, in the case of group C (Supporting Information Figure S4), we observed the occurrence of male CPg‐094 and the females CPg‐010 and CPg‐115 since 2011. Later in 2013 they became connected through newly collected individuals CPg‐128 and CPg‐134. In the case of groups E (Supporting Information Figure S5), any plausible family history was less clear because CPg‐032, the male with the largest number of connecting edges, was sampled only once and the sampling was scarce in the period 2007–2011.

### 3.4 SIVgor evolution

A total of 38 WLG were SIVgor+ (18 females and 20 males) and seven individuals were potentially seroconverters (CPg‐032, CPg‐036, CPg‐041, CPg‐066, CPg‐094, CPg‐115 and CPg‐128. Three individuals sero‐reverted CPg‐003 and CPg‐009 tested positive once but all subsequent samples resulted negative. CPg‐065 was positive in at least of the samples collected during 2010, 2011 and 2012, but tested negative in the samples collected during 2015. Also, CPg‐019 was positive in 2010 but not in earlier (2007) or later samples (2012–2013).

We obtained partial *gp41* gene sequences from 25 genotyped gorillas and partial *pol* gene sequences for 16 of them. The models with the higher log marginal likelihood using either stepping stone sampling or path sampling were a strict molecular clock model and an exponential demographic model (Table S1). In all cases, we obtained “overwhelming” (2 log_e_ Bayes factors [BF] >10) support for strict clocks over relaxed ones (Kass & Raftery*,* 1995). For *gp41,* the exponential model support was “Positive” (2 log_e_ BF 2‐6) over the remaining parametric models and “Strong” (2 log_e_ 6.7) over the nonparametric Skyride; weak support was observed for *pol* when comparing the exponential model to the remaining parametric ones (2 log_e_ BF ~ 1.7) and “Positive” (2 log_e_ BF 3.1) when compared to the Skyride. For *pol*, the inferred time to the most common recent ancestor (TMRCA) was around 1970 (95% HPD interval 1938–1993), the evolutionary rate was 1.27 × 10^−3^ (95% HPD interval 5.06 × 10^−4^ to 2.06 × 10^−3^) and the exponential growth rate was 0.039 per year. For *env*, the inferred TMRCA was around 1964 (95% HPD interval 1940–1984), the evolutionary rate was 5.02 × 10^−3^ (95% HPD interval 2.8 × 10^−3^ to 7.1 × 10^−3^) and the exponential growth rate was 0.033 per year.

**Figure 4:**
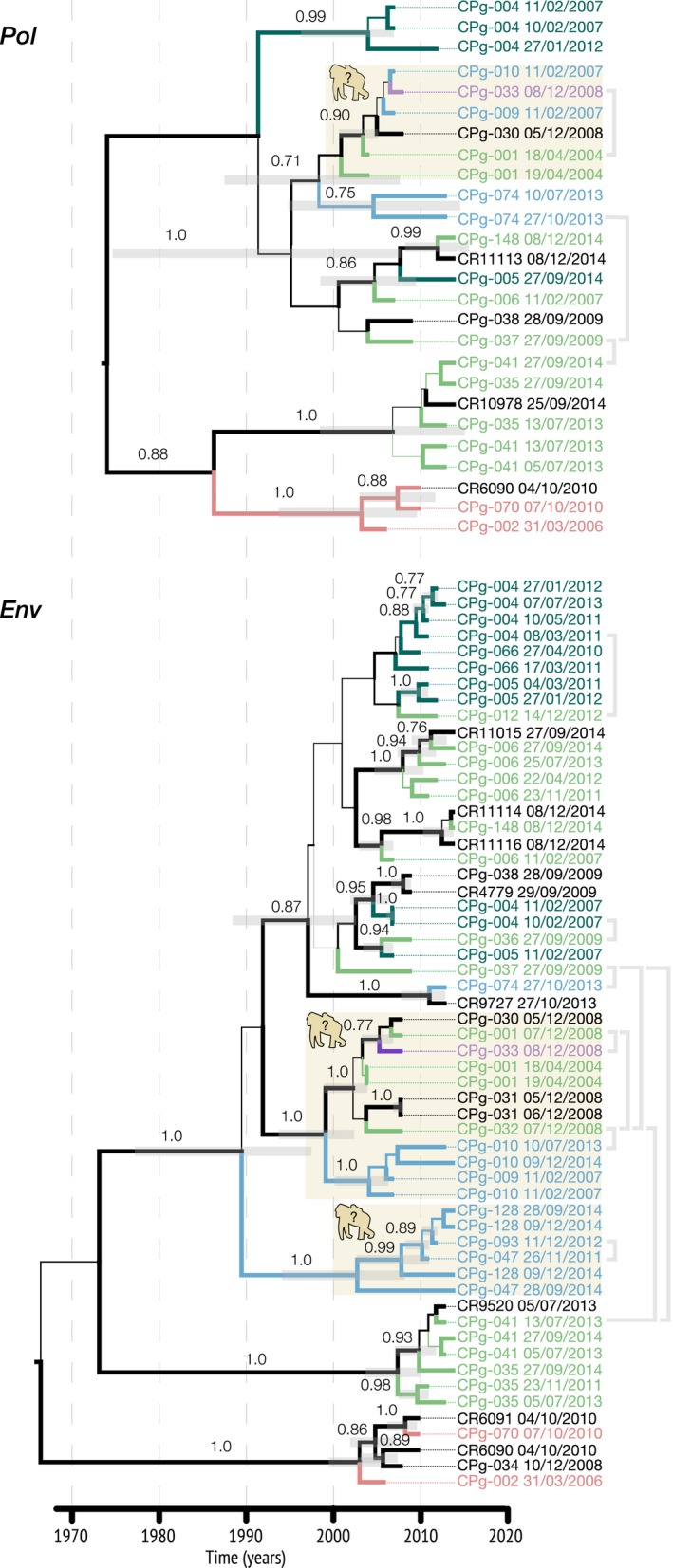
Maximum clade credibility gene trees of SIVgor. (a) Partial pol. (b) partial env. Individuals are coloured according to the groups in Figure 2.; individuals with group uncertain are grey coloured. Line width by posterior probability. Node bars and numerical values are showed for clades with high posterior probability (>0.75). Root bar removed for clarity. Grey brackets in the right side of the trees represent pair‐wise relatedness estimated by any of the metrics (Wang, Maximum Likelihood of Bayesian)—The link was done with the topologically nearest sample when multiple samples per individual were available. The gorilla silhouettes indicate the clades were individuals with apparent vertical transmission fell

Figure 4 shows that the phylogenetic clades were not monophyletic by group but there was some degree of correspondence: For example, individuals CPg‐002 and CPg‐070 (Group F) were observed together in the field and both shared the same viral lineage in both gene trees. Similarly, pairs from Group A (e.g., CPg‐004/CPg‐005), Group C (e.g., CPg‐009/CPg‐010 or CPg‐047/CPg‐093/CPg‐128), and Group E (e.g., pairs CPg001/CPg032, CPg006/CPg148, CPg‐035/CPg‐041) were closely related in the phylogenetic trees.

Sequences from the same individual collected in consecutive days were not identical suggesting a certain degree of intra‐host diversity. Furthermore, we observed that sequences from individuals CPg‐004 and CPg‐005 (who frequently were observed together within group A) sampled in 2007 were distinct from those collected during 2011–2013 in the *env* tree. This pattern could result from either a mixed infection or an exceptionally high viral substitution rate. However, viral samples from individual CPg‐004 collected during 2011–2013 were quite similar, and comparable to the degree of similarity among viral samples collected from other individuals over longer periods of time, that is, CPg‐001 (2004–2008), CPg‐006 (2011–2014), CPg‐010 (2007–2014). Furthermore, we conducted additional analysis constraining the samples from the same individual to be monophyletic and the model selection using path sampling/stepping‐stone sampling preferred the unconstrained model (i.e., it had a higher marginal likelihood and a 2 log_e_ BF of 12.4). These observations favor the mixed infection scenario but we warrant a warning regarding the validity of this conclusion given the uncertainty (i.e., low posterior probability) in the corresponding internal branches.

Concerning the phylogenetic relationships of viral samples from seroconverters Individuals: CPg‐041, CPg‐066, and CPg‐128 grouped with samples from the corresponding same group; CPg‐032 grouped with CPg‐031 whose group identity was uncertain and their closest relative was the clade containing CPg‐001, which belonged to the former's same group; and CPg‐036 closest relative belonged to another group. For the two remaining seroconverters (CPg‐094 and CPg‐115), there were no viral sequence data available.

### 3.5 SIVgor transmission

In order to assess SIVgor transmission we opted for less stringent constraints—in the interindividual's relatedness analysis—to include more pairs where both individuals were SIVgor positive and had SIVgor genetic data. These constraints were as follows: the pairs that had a Wang estimate over the full siblings threshold (0.43 was the data‐inferred relatedness‐threshold for full siblings), the pairs that were discriminated as parent–offspring in the ML analysis and where the log likelihood of the individuals being unrelated was at least three times less than the log likelihood of the parent–offspring, and the pairs with a posterior probability below 0.5 in the Bayesian calculations.

Figure 3 (bottom panel) shows the pairs that had SIVgor genetic data and met the aforementioned requirements. Four pairs had high relatedness values using Wang, ML, and Bayesian calculations: CPg‐010 (group C)/CPg‐032 (group E), CPg‐032 (group E)/CPg‐041 (group E), CPg‐037 (group E)/CPg‐041 (group E), and CPg‐037 (group E)/CPg‐074 (group C). Individuals from the pair CPg‐037/CPg‐074 were relatives in the MSN. Individuals from the pair CPg‐047 (group C)/CPg‐093 (group C) had the highest relatedness value using the Wang method and also were relatives in the MSN. Finally, the trio CPg‐001 (group E)/CPg‐032 (group E)/CPg‐033 (group G) showed high relatedness using the Wang and ML methods and also were relatives in the MSN. All the above instances (represented as gray brackets in Figure 4) represented the most suitable options to identify cases of SIVgor vertical transmission and we investigated if the corresponding viral strains matched in the phylogenetic trees.

Individuals from the pair CPg‐010/CPg‐032 scored high in all relatedness's metrics and fell in a well‐supported clade within the *Env* gene tree along samples CPg‐001, CPg‐009, CPg‐031, and CPg‐033. Moreover, pairs CPg‐001/CPg‐032 and CPg‐001/CPg‐033 had high relatedness values in least one of the metrics and were relatives in the MSN. This group also appeared in the *pol* gene tree. Therefore, it may be the case that at least two individuals among the set CPg‐001/CPg‐031/CPg‐032/CPg‐033 represent a true parent–offspring pair that led to vertical transmission of SIVgor. Similarly, the set CPg‐047/CPg‐093/CPg‐152 scored the highest in the metric of Wang, they were relatives in the MSN and their SIVgor strains of CPg‐047/CPg‐093 were closely related within the *env* gene tree and thus denote another example. Individuals CPg‐032, CPg‐033 CPg‐093, and CPg‐152 were identified later than the remaining individuals in their corresponding set and they were also documented once. Altogether, these observations underscore two instances for which vertical transmission could not be excluded. By contrast, the pairs CPg‐037/CPg‐074, CPg‐037/CPg‐041, CPg‐032/CPg‐041 scored high in all relatedness metrics and some of them were relatives in the MSN but their respective SIVgor strains fell apart in both gene trees. Therefore, in these instances the mots likely scenario is the horizontal transmission of SIVgor.

## 4 DISCUSSION

The present study provides new insights into the ecology of unhabituated gorillas from the Campo‐Ma'an National Park and into SIVgor evolution. Our findings expanded those of et al. Etienne et al. (2011): firstly, we not only included data from a longer observation period (2004–2014) but also doubled the number of microsatellite markers for each individual allowing for a more thorough characterization of the wildlife population. Secondly, with the increased number of observations we provided a more detailed and accurate depiction of the social structure of the groups. Finally, we refined both genetic and phylogenetic analyses using state‐of‐the‐art maximum‐likelihood and Bayesian approaches. The long‐term follow‐up allowed us to document an individual over an a span of 10 years and to re‐capture several other individuals providing room for further development of noninvasive sampling of free‐ranging apes. Our work relied on the sampling of fecal specimens and our collection procedures was chosen in a way that preserved RNA and DNA in stool and prevented nucleic acid degradation. As a result, we succeeded in genotyping individuals using amplified microsatellite data and we also succeeded in amplifying and sequencing SIVgor genetic data from some of the seropositive individuals. Overall, we validated the usefulness of noninvasive sampling to study wild primate populations and infectious diseases.

Western lowland gorillas live in groups (also known as troops) that typically include one adult silverback, multiple females, and their offspring (Breuer et al., 2009; Gatti, Levrero, Menard, & Gautier‐Hion, 2004; Parnell, 2002; Robbins et al., 2004). Using the geographical coordinates and a simple algorithm, we recognized at least six different groups with the largest group having a maximum of 14 individuals at the same time. These results are comparable to those obtained in the studies reviewed by Yamagiwa et al. (five localities in Gabón and the Republic of the Congo) (Yamagiwa, Kahekwa, & Basabose, 2003) where they reported that the group size ranged between 7 and 10 individuals and that the maximum group size ranged between 10 and 18 individuals.

The groups proposed by our study did not exhibit any clear spatial distribution at the current geographical scale. Some groups were only observed once or only during a couple of years. The infrequent sampling of some groups may be the result of sampling efforts: our field teams prioritized sites where gorillas were already spotted in the past. Groups of western lowland gorillas have an annual home range of 7–14 km^2^ (Bermejo, 2004; Yamagiwa et al., 2003), and it is possible that some groups roamed to other sites of the park in the following years. Females predominated in most groups but there were several instances where more than one male was present in other groups. Multi‐male and all‐male groups are suggested to be rare in western lowland gorillas, when compared to mountain gorillas, where male emigration is lower (Davenport, 2008; Gatti et al., 2004; Parnell, 2002). Multi‐male groups are the outcome of maturing male offspring that do not emigrate out of the group but in our case we cannot discard the possibility of these males being infants or juveniles; in most cases, the males were related. Western lowland gorillas can also be found in nonbreeding, all‐male groups that can last for several years, although they are subject to frequent membership changes due to male migrations (Gatti et al., 2004).

Gorillas are among the relatively few mammal species for which both sexes usually disperse from their natal groups (Stoinski, Perdue, & Legg, 2009; Stokes, Parnell, & Olejniczak, 2003). Consequently, the composition and stability of gorilla groups varies greatly over time. Our MSN reflect such variation providing no evidence of population structure at this scale and instead suggest high gene flow. At higher scales, Funfstuck et al. (2014) found three significantly differentiated genetic clusters of western lowland gorillas (the whole studied region covered approximately 37,000 km^2^ and included sampling sites in national parks from Cameroon, Central African Republic, and the Republic of Congo): the boundaries of the genetic groups coincided with courses of major rivers suggesting that major landforms drive the patterns of genetic differentiation.

Understanding wild primate group dynamics is critical to anticipate the demographic consequences of human activities, to measure the impact of disease outbreaks and to identify other drivers of demographical change. Interactions between lowland gorillas groups occur more frequently than they do in mountain gorillas and are often nonaggressive (Bradley, Doran‐Sheehy, Lukas, Boesch, & Vigilant, 2004; Doran‐Sheehy, Greer, Mongo, & Schwindt, 2004). Bradley et al. (2004) underscored that such nonaggressive behaviour is attributed to male's kinship networks across groups (related males leading groups that range in the same area) and that this kind of encounters facilitates high rates of migration of females. Although in this study we did not find a clear male's kinship networks across groups, we provided additional evidence of migration based on SIV linage associations. We evidenced the same SIVgor strain circulating in different groups and correspondingly, different lineages within the same group. The lack of a distinctive viral lineage for every group underscores that there is not major viral transmission barrier for the spread of SIV and that pathogens can rapidly spread between groups.

Simian immunodeficiency virus vertical transmission (from mother to infant) is argued to be less frequent than horizontal transmission (between individuals, through wounds, and sexual contact). The junction of our genetic and phylogenetic findings provides evidence of SIVgor transmission among close relatives in two different sets of gorillas. Closely related viruses have being observed in a presumed parent–offspring pair of chimpanzees suggesting vertical transmission (Keele et al., 2009). Moreover, correlation between SIV infection and maternal kinship that does not involve vertical transmission has been documented in Mandrills (through allogrooming, wound care, or the inheritance of genetic determinants of susceptibility to SIV among others) (Fouchet et al., 2012) and in one infant chimpanzee through breast milk transmission (Keele et al., 2009). In any of the two sets of gorillas with probable vertical transmission, we observed that the individuals who were sampled last were also observed only once but we do not have enough evidence to rule out that this is due to increased mortality rather than to chance. In the same vein, it is generally considered that SIVs do not generally cause acquired immunodeficiency syndrome in their natural hosts but Pandrea, Silvestri, and Apetrei (2009) argued that SIV infection should be regarded as persistently nonprogressive SIV infections (Klatt, Silvestri, & Hirsch, 2012; Pandrea et al., 2009). For example, SIVcpz infection has a substantial negative impact on the health, reproduction, and lifespan of individuals in the wild (Keele et al., 2009). The likelihood of occasional progression to disease emphasizes the importance of monitoring gorillas and characterizing SIVgor diversity and pathogenesis. The later is of paramount significance because even though western lowland gorillas are the most numerous and widespread of all gorilla subspecies, hunting, logging, and disease are deeply threatening their populations.

Modern‐day SIV diversity was generated by extensive recombination and cross‐species transmission (Bell & Bedford, 2017). The simian immunodeficiency virus of chimpanzees (SIVcpz) was transmitted once to gorillas generating SIVgor, which was in turn transmitted twice to humans generating two distinct groups of HIV‐1 (D'Arc et al., 2015; Van Heuverswyn et al., 2006). The complex evolutionary history of SIVs involving a long chain of host switches and recombination events illustrates the importance of characterizing modern day viral diversity. Simian immunodeficiency virus genetic characterization is scarce because to identify and monitor infected gorillas in the wild poses a big challenge. Our viral demographic analysis using genetic data suggested that SIV in the Campo‐Ma'an National Park experiences a demographical exponential growth. The exponential growth coupled with a strict molecular clock suggests steady transmission and diversification of viral lineages over time. The estimated growth rates were similar for the two genetic regions and were in the same order of magnitude as those of HIV group M in the prepandemic period (Faria et al., 2014; Villabona Arenas et al., 2017). The upper bound of the tMRCA for SIVgor in this particular region was around the late 1930s in line with the idea that SIVs are in general young lentiviral lineages (Wertheim & Worobey, 2009). Nonetheless, these inferences need to be taken with some caution. Small regions might lack sufficient signal to draw accurate inference about tMRCAs, although in our case both genes had uniform priors placed in their respective root parameters and the estimated TMRCAs were in agreement. Also, we included sequences from the same individual sampled at different time points to better characterize likely instances of transmission. However, within host variation may impact viral growth rate estimates and although we ranked competing models and used the best fit to the empirical data the latter do not necessarily reflect the overall population dynamics.

Our study has limitations. Our sampling relied on the opportunistic collection of samples and therefore we cannot exclude that some regions of the Campo‐Ma'an National Park and some groups were missed. Although individuals from both sexes frequently disperse when reaching sexual maturity (Stoinski et al., 2009; Stokes et al., 2003) with not quantitative sex‐bias in migration (Funfstuck et al., 2014), our clustering algorithm does not consider migration of individuals between groups because this requires the frequent recapture of the same individuals and a more thorough sampling. Previous studies showed that the mean distance between individuals in zoo‐housed lowland gorilla groups was in average 23 m while wild mountain gorillas usually stay within 10 m of other members (Kurtycz, Shender, & Ross, 2014; Juichi Yamagiwa, 1983). Moreover, we documented four cases of one individual being sampled twice on the same day and the averaged distance between paired samples was 200 m. Consequently, our clustering distance values were chosen to reflect this but we cannot exclude that the largest groups may represent multiple groups that frequently exchanged individuals, that merged, or whose action range overlapped prior to the time of sampling (the home range of gorillas extensively overlap with those of neighboring groups and they have a large mean day journey length, between 1,100 and 2,600 m (Yamagiwa et al., 2003). However, in addition to using multiple distance cutoffs, we also removed observations in sensitivity analyses and the resulting groups represent the best division in terms of occurrence and field research. Finally, we were not able to distinguish between adults and nonadults, to genotype all samples or to amplify SIVgor from all seropositive samples. Although we have characterized nearly complete SIV genomes from some fecal samples (D'Arc et al., 2015) we succeeded more often with smaller genetic fragments.

Despite all the aforementioned issues, our results validate a noninvasive approach to the recovery of otherwise unobtainable information directly related to the ecology of gorillas and of infectious diseases in the wilderness. In the same vein, this approach could allow to survey the microbiome and the virome using metagenomics. For example, D'arc et al. provided evidence of association of specific mammalian viral families and SIVgor in a dysbiosis context (D'Arc et al., 2018). Thus, these studies provide a baseline to identify markers associated with pathogenic conditions.

In conclusion, given the critically endangered status of western lowland gorillas, a noninvasive sampling represents a viable approach to study wild population and disease and the findings derived from it contribute to the prospective planning for better monitoring and conservation and also contribute to a better understanding of SIVgor evolution and transmission.

## CONFLICT OF INTEREST

None declared.

## AUTHOR CONTRIBUTIONS

CJVA designed and performed data analysis, interpreted the data and wrote the manuscript; AA, AE, MD performed laboratory and data analysis; EMN coordinated field studies; MP designed analysis, interpreted the data, and wrote the manuscript; all authors discussed the results and implications.

## DATA ACCESSIBILITY

New sequences were deposited in GenBank under accessions MH646552‐MH646620. Microsatellite genotypes are available as Supporting Information Table S2.
