# Supplementary figures and images for: Noninvasive western lowland gorilla's health monitoring: A decade of simian immunodeficiency virus surveillance in southern Cameroon
Source: Ecol Evol. 2018 Oct 25;8(22):10698–710. doi: 10.1002/ece3.4478 (PMC6262910; doi:10.1002/ece3.4478)

A

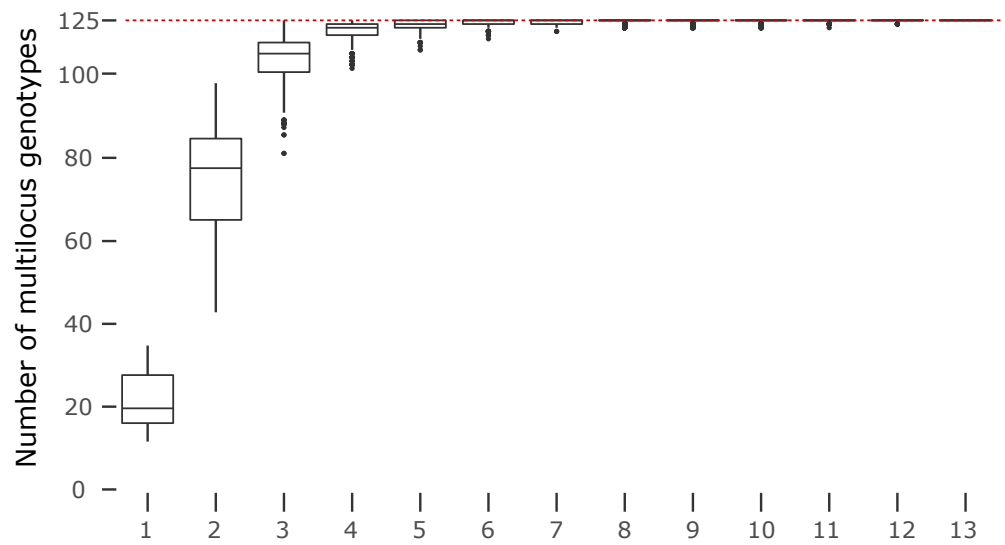

B

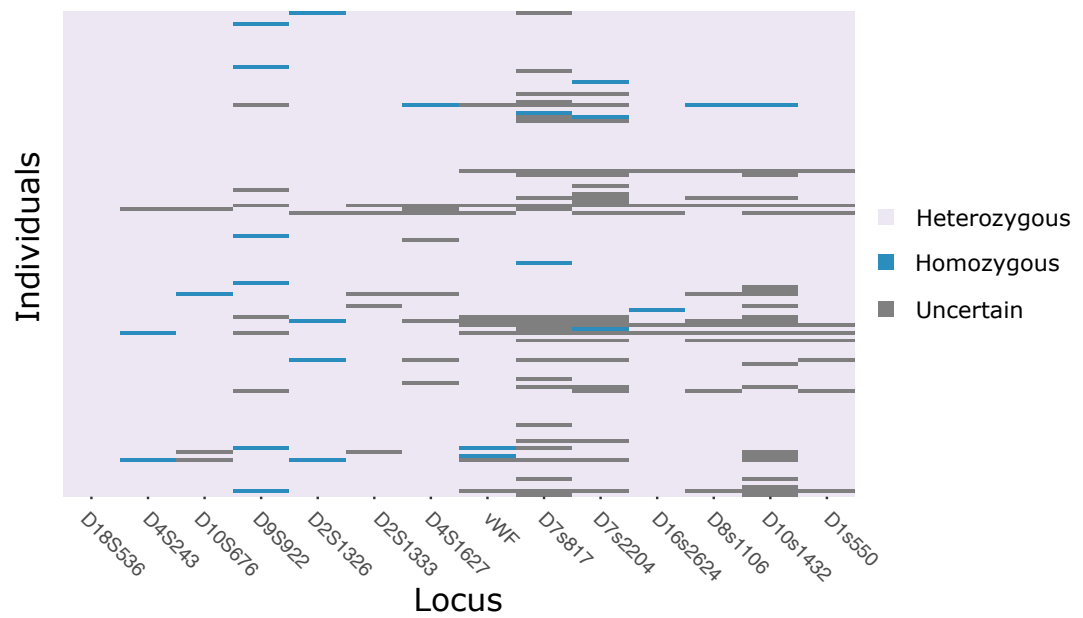

C

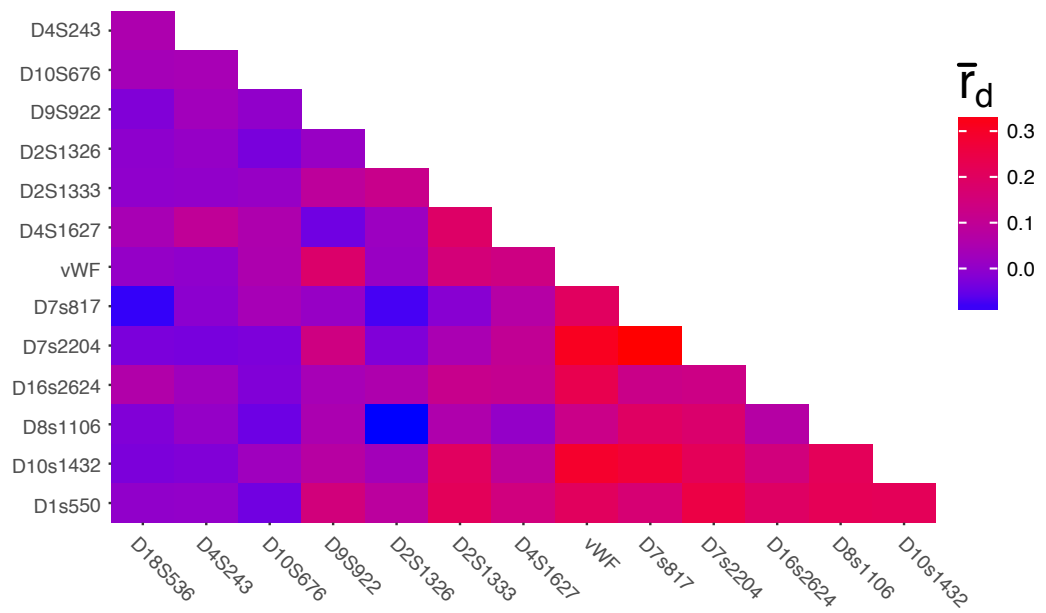

Supplement: Supplementary file 1 [file ECE3-8-10698-s001.pdf]

Group A

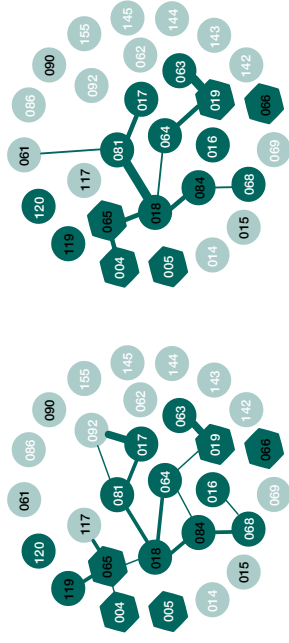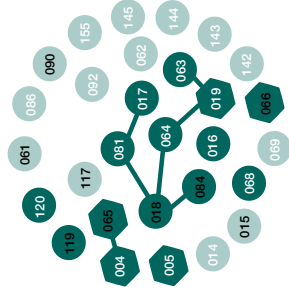

Wang ML

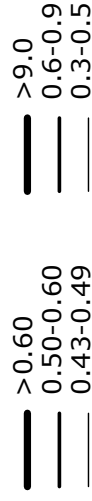

Bayes

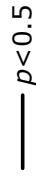

Group C

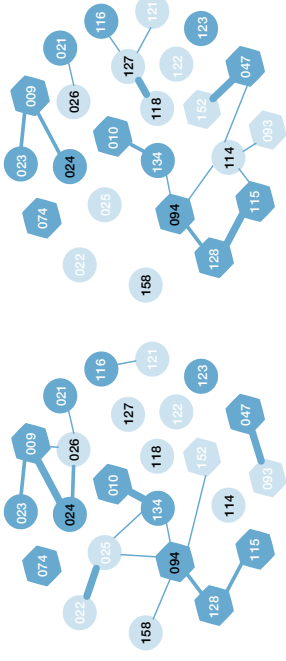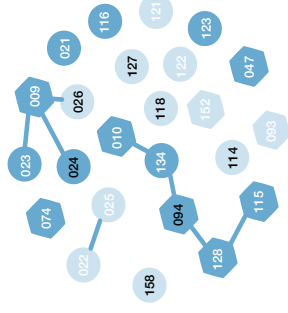

Group E

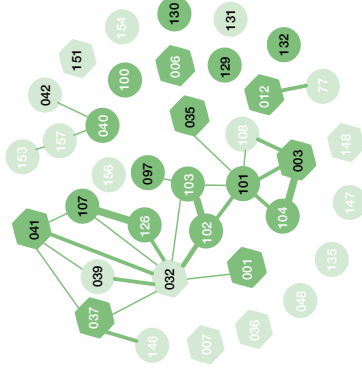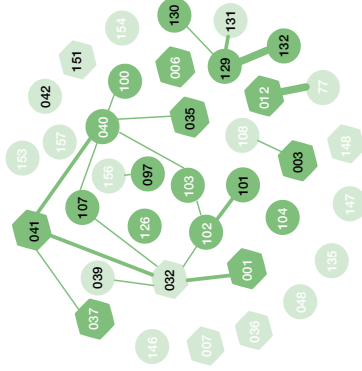

Group G

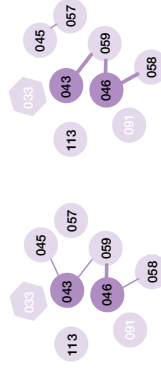

Group B

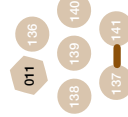

None

None

Supplement: Supplementary file 2 [file ECE3-8-10698-s002.pdf]

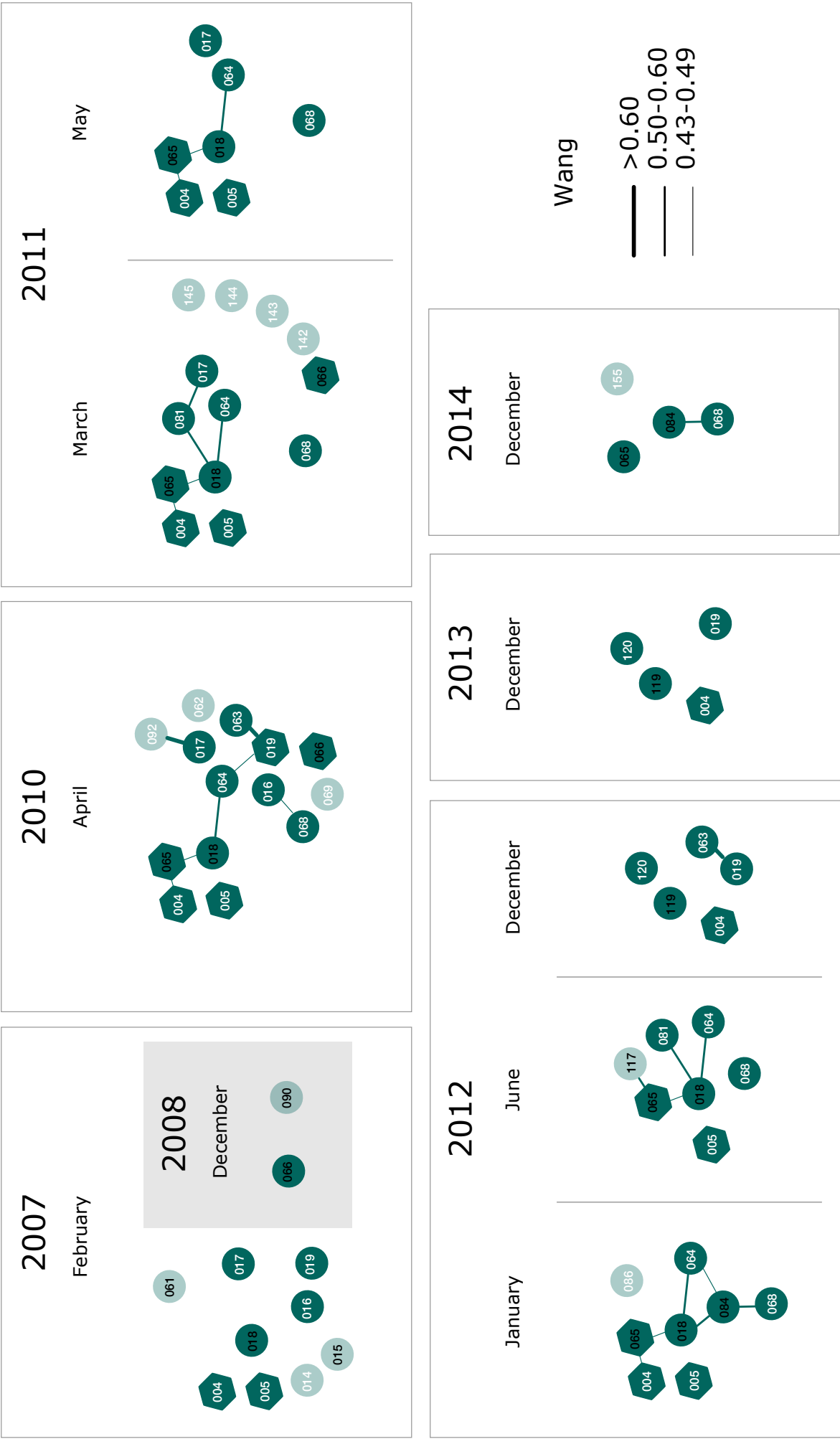

Supplement: Supplementary file 3 [file ECE3-8-10698-s003.pdf]

2007

February

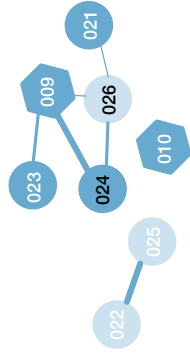

2011

November

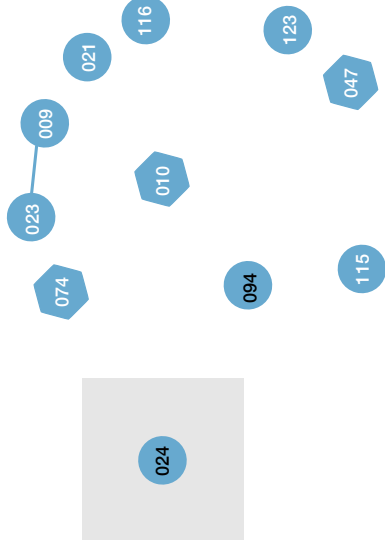

2012

December

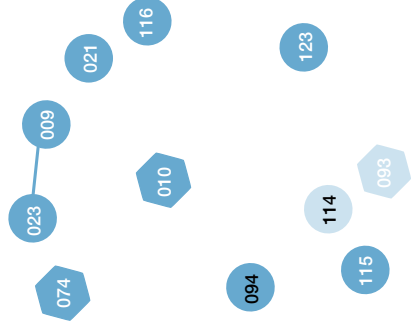

2013

July

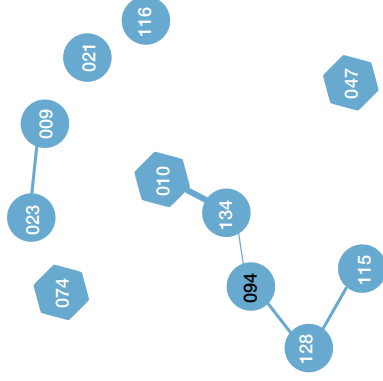

October

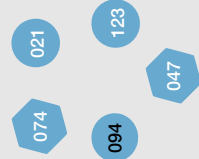

2014

February

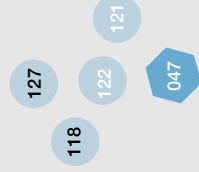

September

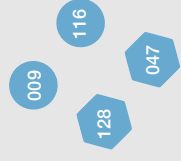

December

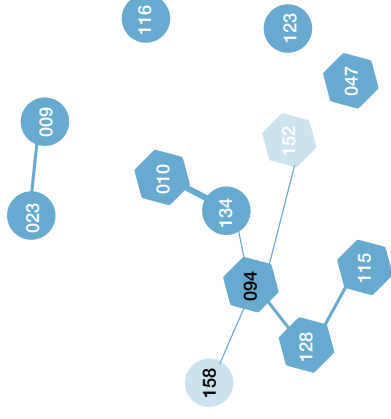

Wang

— >0.60

— 0.50-0.60

— 0.43-0.49

Supplement: Supplementary file 4 [file ECE3-8-10698-s004.pdf]

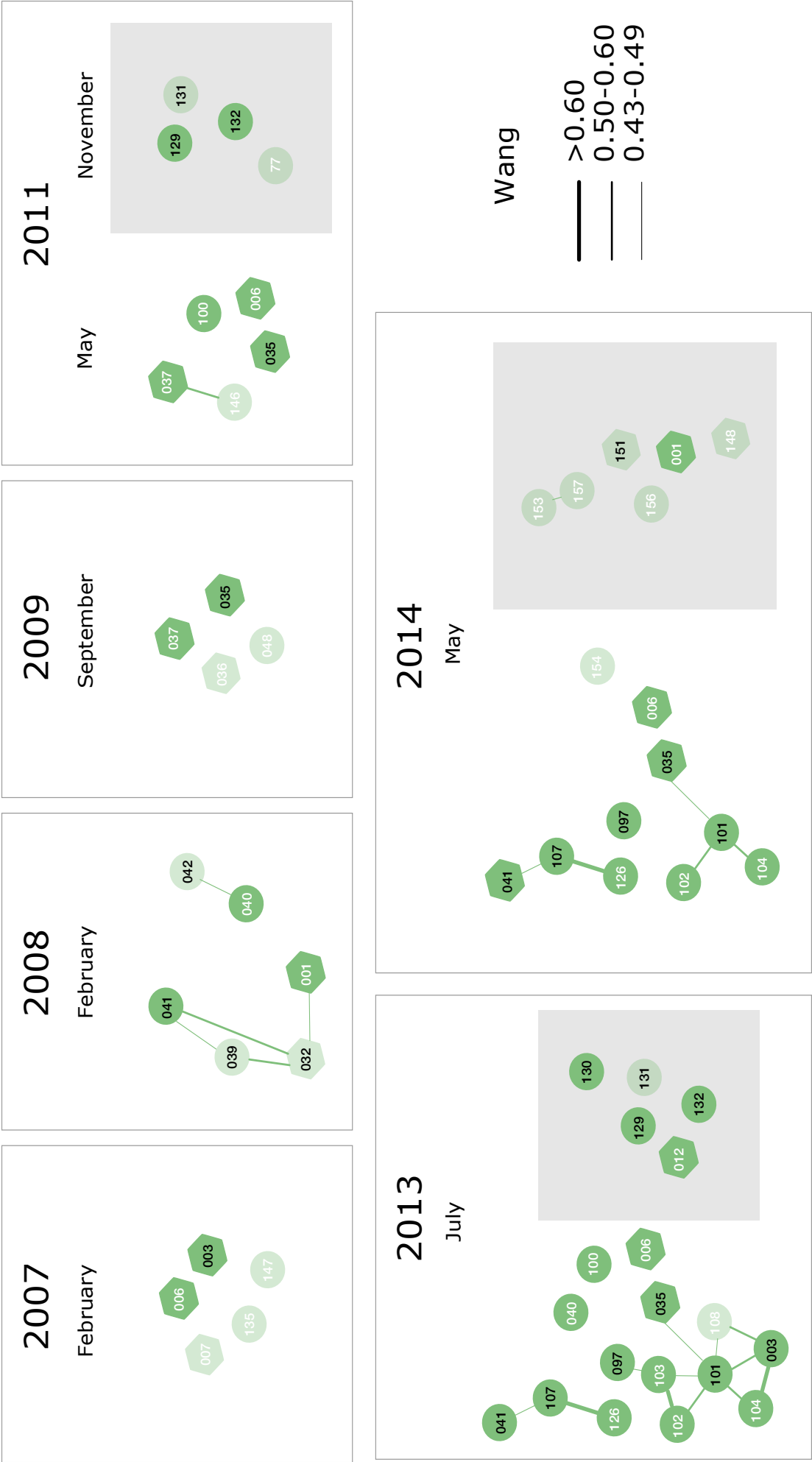

Supplement: Supplementary file 5 [file ECE3-8-10698-s005.pdf]

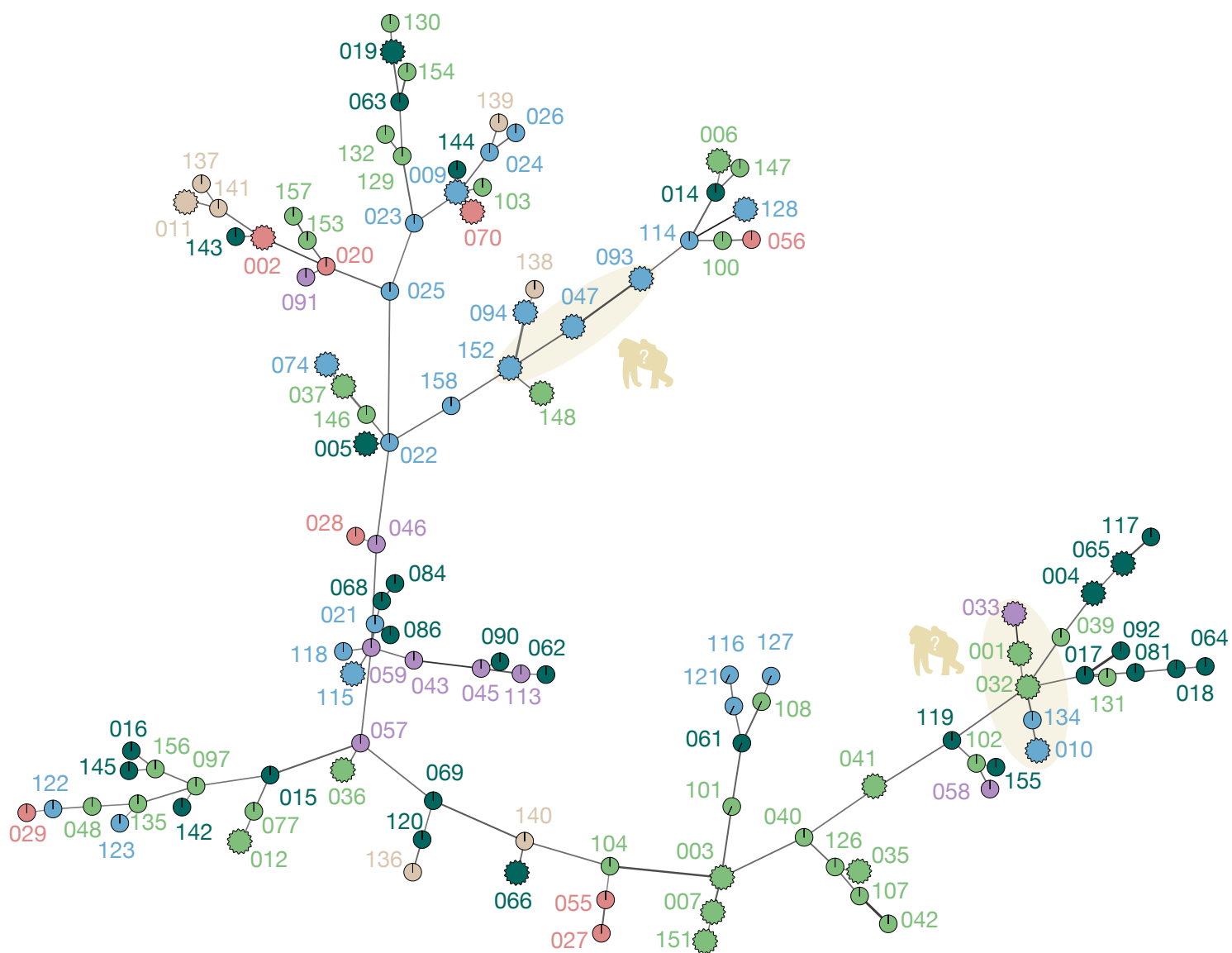

Supplement: Supplementary file 6 [file ECE3-8-10698-s006.pdf]
