# Supplementary material for: Noninvasive western lowland gorilla's health monitoring: A decade of simian immunodeficiency virus surveillance in southern Cameroon
Source: Ecol Evol. 2018 Oct 25;8(22):10698–710. doi: 10.1002/ece3.4478 (PMC6262910; doi:10.1002/ece3.4478)
Supplement: Supplementary file 7 [file ECE3-8-10698-s007.docx]

**Table S1. Model comparison using path sampling (PS) and stepping stone (SS) marginal Likelihood estimates.** Bayes factors were calculated following the formula in the top-left corner. Values in brackets favour models in the top axis, values without bracket values favour models in the left axis. Models with the highest marginal likelihood estimate in bold. Con, constant. Exp, Exponential. Log, Logistic. BS, non-parametric Bayesian Skyride.

|  |  |  |  |  |  |  |  |  |  |  |  |  |
| --- | --- | --- | --- | --- | --- | --- | --- | --- | --- | --- | --- | --- |
| **2LN(M*y*-M*x*)** | | | | | **Relaxed** | | | | **Strict** | | | |
|  |  |  |  |  | **Con** | **Exp** | **Log** | **BS** | **Con** | **Exp** | **Log** | **BS** |
| Gene | **Clock** | **Tree Prior** | **PS** | **SS** |  |  |  |  |  |  |  |  |
| *gP41* | **Relaxed** | **Constant** | -3807.5 | -3808.21 |  |  |  |  |  |  |  |  |
|  |  | **Exponential** | -3807.2 | -3807.64 | 0.00 |  |  |  |  |  |  |  |
|  |  | **Logistic** | -3801.9 | -3802.32 | 3.55 | 3.34 |  |  |  |  |  |  |
|  |  | **Bayesian Skyride** | -3796.3 | -3791.15 | 5.67 | 5.61 | 4.83 |  |  |  |  |  |
|  | **Strict** | **Constant** | -2827.6 | -2826.92 | 13.78 | 13.78 | 13.77 | 13.74 |  |  |  |  |
|  |  | **Exponential** | **-2817.3** | **-2818.43** | 13.79 | 13.79 | 13.78 | 13.76 | 4.28 |  |  |  |
|  |  | **Logistic** | -2822.3 | -2823.06 | 13.79 | 13.78 | 13.77 | 13.75 | 2.70 | [3.06] |  |  |
|  |  | **Bayesian Skyride** | -2846 | -2846.88 | 13.74 | 13.74 | 13.72 | 13.70 | [5.98] | [6.70] | [6.71] |  |
| *pol* | **Relaxed** | **Constant** | -1250.4 | -1250.74 |  |  |  |  |  |  |  |  |
|  |  | **Exponential** | -1245.9 | -1245.71 | 3.23 |  |  |  |  |  |  |  |
|  |  | **Logistic** | -1249.2 | -1249.01 | 1.09 | [2.38] |  |  |  |  |  |  |
|  |  | **Bayesian Skyride** | -1251.8 | -1251.71 | 0.00 | [3.58] | [1.99] |  |  |  |  |  |
|  | **Strict** | **Constant** | -898.6 | -898.03 | 11.73 | 11.70 | 11.72 | 11.74 |  |  |  |  |
|  |  | **Exponential** | **-895.59** | **-895.71** | 11.74 | 11.72 | 11.73 | 11.75 | 1.68 |  |  |  |
|  |  | **Logistic** | -897.56 | -898.14 | 11.73 | 11.70 | 11.72 | 11.74 | [4.41] | [1.77] |  |  |
|  |  | **Bayesian Skyride** | -901.31 | -900.45 | 11.72 | 11.69 | 11.71 | 11.72 | [1.77] | [3.11] | [1.67] |  |
