# Supplementary material for: Noninvasive western lowland gorilla's health monitoring: A decade of simian immunodeficiency virus surveillance in southern Cameroon
Source: Ecol Evol. 2018 Oct 25;8(22):10698–710. doi: 10.1002/ece3.4478 (PMC6262910; doi:10.1002/ece3.4478)
Supplement: Supplementary file 8 [file ECE3-8-10698-s008.docx]

**Table S2. Microsatellite genotypes for western lowland gorilla (Gorilla gorilla gorilla) from the Campo-Ma'an National Park identified between February 2006 and December 2014**

|  | **D18S536** |  | **D4S243** |  | **D10S676** |  | **D9S922** |  | **D2S1326** |  | **D2S1333** |  | **D4S1627** |  | **vWF** |  | **D7s817** |  | **D7s2204** |  | **D16s2624** |  | **D8s1106** |  | **D10s1432** |  | **D1s550** |  |
| --- | --- | --- | --- | --- | --- | --- | --- | --- | --- | --- | --- | --- | --- | --- | --- | --- | --- | --- | --- | --- | --- | --- | --- | --- | --- | --- | --- | --- |
| **CPg-ID001** | 142 | 146 | 185 | 193 | 180 | 192 | 280 | 280 | 251 | 0 | 298 | 318 | 234 | 274 | 140 | 144 | 0 | 0 | 244 | 244 | 137 | 145 | 144 | 144 | 166 | 166 | 183 | 195 |
| **CPg-ID002** | 146 | 154 | 181 | 193 | 196 | 200 | 276 | 280 | 255 | 267 | 298 | 298 | 234 | 242 | 160 | 160 | 188 | 188 | 244 | 248 | 137 | 137 | 144 | 144 | 170 | 170 | 179 | 183 |
| **CPg-ID003** | 146 | 150 | 181 | 185 | 196 | 200 | 272 | 280 | 251 | 271 | 314 | 334 | 234 | 238 | 152 | 152 | 192 | 192 | 0 | 0 | 137 | 137 | 140 | 144 | 162 | 166 | 171 | 183 |
| **CPg-ID004** | 146 | 146 | 185 | 193 | 196 | 200 | 0 | 0 | 267 | 275 | 322 | 334 | 234 | 274 | 152 | 156 | 176 | 176 | 232 | 244 | 137 | 141 | 140 | 144 | 158 | 166 | 183 | 191 |
| **CPg-ID005** | 146 | 150 | 177 | 185 | 192 | 200 | 260 | 280 | 251 | 267 | 322 | 326 | 234 | 246 | 148 | 156 | 176 | 188 | 244 | 244 | 145 | 145 | 140 | 144 | 166 | 170 | 183 | 187 |
| **CPg-ID006** | 146 | 146 | 177 | 181 | 196 | 200 | 264 | 280 | 267 | 275 | 322 | 334 | 242 | 246 | 152 | 152 | 180 | 184 | 244 | 248 | 141 | 141 | 144 | 148 | 174 | 178 | 183 | 187 |
| **CPg-ID007** | 146 | 150 | 185 | 185 | 196 | 200 | 0 | 0 | 251 | 287 | 0 | 0 | 0 | 0 | 0 | 0 | 0 | 0 | 0 | 0 | 0 | 0 | 0 | 0 | 0 | 0 | 0 | 0 |
| **CPg-ID009** | 146 | 146 | 181 | 189 | 192 | 200 | 276 | 280 | 251 | 271 | 318 | 334 | 242 | 242 | 148 | 152 | 184 | 192 | 244 | 248 | 141 | 145 | 140 | 148 | 166 | 166 | 179 | 183 |
| **CPg-ID010** | 146 | 154 | 173 | 193 | 180 | 180 | 276 | 280 | 255 | 271 | 298 | 322 | 230 | 234 | 148 | 152 | 188 | 196 | 232 | 244 | 137 | 137 | 140 | 144 | 166 | 178 | 179 | 183 |
| **CPg-ID011** | 146 | 150 | 181 | 189 | 196 | 200 | 264 | 280 | 263 | 267 | 314 | 318 | 234 | 274 | 156 | 160 | 184 | 184 | 236 | 240 | 141 | 145 | 144 | 144 | 166 | 178 | 179 | 195 |
| **CPg-ID012** | 150 | 154 | 185 | 185 | 188 | 196 | 0 | 280 | 267 | 275 | 298 | 306 | 242 | 274 | 140 | 156 | 176 | 176 | 244 | 248 | 137 | 141 | 140 | 148 | 158 | 166 | 179 | 183 |
| **CPg-ID014** | 146 | 146 | 0 | 0 | 0 | 0 | 268 | 276 | 263 | 275 | 330 | 330 | 0 | 0 | 152 | 152 | 0 | 0 | 244 | 244 | 141 | 145 | 144 | 144 | 178 | 178 | 179 | 191 |
| **CPg-ID015** | 146 | 150 | 185 | 193 | 192 | 192 | 268 | 280 | 0 | 0 | 0 | 0 | 0 | 0 | 0 | 0 | 176 | 176 | 0 | 0 | 0 | 0 | 152 | 152 | 0 | 0 | 0 | 0 |
| **CPg-ID016** | 146 | 154 | 173 | 177 | 196 | 200 | 280 | 280 | 267 | 267 | 334 | 334 | 230 | 230 | 148 | 152 | 176 | 188 | 240 | 244 | 141 | 145 | 140 | 144 | 166 | 170 | 179 | 183 |
| **CPg-ID017** | 146 | 154 | 193 | 193 | 192 | 196 | 280 | 284 | 251 | 271 | 314 | 326 | 242 | 274 | 148 | 156 | 192 | 196 | 248 | 248 | 137 | 137 | 140 | 144 | 166 | 166 | 183 | 183 |
| **CPg-ID018** | 146 | 154 | 193 | 193 | 196 | 200 | 280 | 280 | 247 | 251 | 314 | 322 | 234 | 246 | 148 | 152 | 188 | 188 | 244 | 248 | 137 | 145 | 144 | 144 | 162 | 162 | 183 | 183 |
| **CPg-ID019** | 146 | 154 | 181 | 185 | 192 | 196 | 264 | 264 | 255 | 271 | 318 | 318 | 242 | 242 | 140 | 148 | 180 | 184 | 236 | 244 | 137 | 141 | 144 | 144 | 162 | 162 | 171 | 187 |
| **CPg-ID020** | 146 | 154 | 173 | 181 | 196 | 200 | 276 | 280 | 255 | 267 | 298 | 318 | 234 | 242 | 0 | 0 | 0 | 0 | 0 | 0 | 0 | 0 | 0 | 0 | 0 | 0 | 0 | 0 |
| **CPg-ID021** | 154 | 154 | 185 | 185 | 192 | 200 | 254 | 0 | 251 | 271 | 334 | 334 | 230 | 274 | 152 | 156 | 188 | 196 | 240 | 244 | 137 | 137 | 140 | 152 | 158 | 170 | 183 | 183 |
| **CPg-ID022** | 146 | 154 | 181 | 185 | 192 | 196 | 268 | 280 | 251 | 267 | 296 | 318 | 0 | 0 | 152 | 152 | 176 | 188 | 244 | 248 | 137 | 145 | 140 | 148 | 158 | 166 | 179 | 183 |
| **CPg-ID023** | 146 | 154 | 181 | 189 | 192 | 200 | 268 | 276 | 271 | 275 | 310 | 318 | 234 | 242 | 148 | 152 | 176 | 184 | 244 | 248 | 137 | 145 | 144 | 148 | 166 | 166 | 179 | 179 |
| **CPg-ID024** | 146 | 146 | 189 | 193 | 180 | 192 | 268 | 280 | 251 | 275 | 318 | 334 | 230 | 242 | 152 | 152 | 184 | 192 | 244 | 248 | 137 | 141 | 140 | 140 | 166 | 166 | 183 | 183 |
| **CPg-ID025** | 146 | 154 | 181 | 185 | 192 | 196 | 268 | 280 | 251 | 267 | 298 | 318 | 230 | 242 | 152 | 152 | 176 | 188 | 244 | 248 | 137 | 141 | 140 | 148 | 158 | 166 | 179 | 183 |
| **CPg-ID026** | 146 | 154 | 185 | 189 | 180 | 200 | 268 | 280 | 255 | 271 | 334 | 334 | 230 | 242 | 152 | 156 | 184 | 196 | 244 | 248 | 137 | 141 | 140 | 140 | 158 | 166 | 183 | 183 |
| **CPg-ID027** | 150 | 150 | 181 | 181 | 188 | 192 | 264 | 280 | 251 | 255 | 318 | 326 | 242 | 278 | 152 | 156 | 176 | 188 | 240 | 244 | 145 | 145 | 144 | 144 | 162 | 162 | 171 | 171 |
| **CPg-ID028** | 146 | 150 | 173 | 173 | 196 | 200 | 276 | 280 | 251 | 275 | 306 | 334 | 234 | 246 | 152 | 160 | 176 | 180 | 240 | 244 | 137 | 141 | 144 | 148 | 158 | 162 | 175 | 183 |
| **CPg-ID029** | 146 | 154 | 173 | 193 | 192 | 196 | 280 | 288 | 251 | 255 | 326 | 334 | 242 | 246 | 148 | 152 | 180 | 184 | 252 | 252 | 137 | 137 | 148 | 152 | 158 | 170 | 187 | 187 |
| **CPg-ID030** | 146 | 146 | 185 | 189 | 180 | 196 | 268 | 272 | 267 | 271 | 0 | 0 | 234 | 242 | 152 | 152 | 188 | 188 | 244 | 248 | 137 | 145 | 148 | 152 | 0 | 0 | 183 | 187 |
| **CPg-ID031** | 146 | 146 | 173 | 189 | 180 | 196 | 276 | 280 | 255 | 267 | 318 | 322 | 242 | 274 | 152 | 152 | 180 | 192 | 244 | 252 | 0 | 141 | 148 | 152 | 166 | 170 | 179 | 187 |
| **CPg-ID032** | 146 | 154 | 193 | 193 | 180 | 192 | 280 | 280 | 251 | 271 | 298 | 334 | 234 | 274 | 144 | 152 | 188 | 196 | 232 | 244 | 137 | 137 | 140 | 144 | 166 | 166 | 183 | 183 |
| **CPg-ID033** | 142 | 146 | 169 | 185 | 180 | 192 | 276 | 280 | 251 | 251 | 298 | 318 | 230 | 234 | 140 | 144 | 192 | 196 | 244 | 244 | 137 | 145 | 144 | 144 | 166 | 166 | 183 | 195 |
| **CPg-ID034** | 146 | 150 | 193 | 193 | 192 | 200 | 264 | 276 | 251 | 255 | 298 | 334 | 230 | 242 | 156 | 156 | 172 | 184 | 248 | 252 | 133 | 137 | 144 | 148 | 162 | 162 | 183 | 183 |
| **CPg-ID035** | 146 | 146 | 177 | 189 | 196 | 200 | 0 | 280 | 255 | 271 | 314 | 334 | 234 | 274 | 144 | 152 | 180 | 192 | 244 | 252 | 133 | 137 | 140 | 144 | 162 | 162 | 183 | 187 |
| **CPg-ID036** | 146 | 154 | 185 | 193 | 192 | 196 | 268 | 276 | 247 | 267 | 322 | 330 | 234 | 242 | 152 | 156 | 176 | 184 | 240 | 244 | 137 | 137 | 144 | 144 | 162 | 166 | 179 | 191 |
| **CPg-ID037** | 146 | 154 | 177 | 193 | 192 | 196 | 264 | 268 | 251 | 267 | 334 | 334 | 234 | 274 | 140 | 152 | 188 | 196 | 240 | 248 | 137 | 145 | 140 | 148 | 158 | 166 | 183 | 183 |
| **CPg-ID038** | 146 | 150 | 185 | 193 | 196 | 200 | 280 | 284 | 267 | 275 | 314 | 322 | 234 | 242 | 144 | 148 | 176 | 176 | 240 | 260 | 137 | 137 | 148 | 148 | 166 | 170 | 179 | 183 |
| **CPg-ID039** | 146 | 154 | 189 | 193 | 180 | 196 | 268 | 280 | 271 | 275 | 318 | 334 | 234 | 270 | 140 | 152 | 176 | 196 | 232 | 244 | 137 | 137 | 140 | 144 | 162 | 166 | 183 | 183 |
| **CPg-ID040** | 146 | 150 | 177 | 181 | 196 | 200 | 0 | 0 | 271 | 271 | 314 | 334 | 234 | 274 | 0 | 0 | 0 | 0 | 0 | 0 | 137 | 145 | 144 | 144 | 0 | 0 | 171 | 183 |
| **CPg-ID041** | 146 | 150 | 177 | 193 | 180 | 196 | 264 | 280 | 251 | 271 | 314 | 334 | 234 | 234 | 144 | 152 | 184 | 196 | 232 | 248 | 137 | 145 | 140 | 144 | 166 | 166 | 171 | 183 |
| **CPg-ID042** | 142 | 146 | 181 | 193 | 180 | 200 | 272 | 280 | 271 | 271 | 334 | 334 | 230 | 234 | 0 | 0 | 0 | 0 | 0 | 0 | 0 | 0 | 0 | 0 | 0 | 0 | 0 | 0 |
| **CPg-ID043** | 150 | 154 | 181 | 185 | 192 | 200 | 268 | 280 | 251 | 251 | 298 | 334 | 230 | 242 | 156 | 156 | 188 | 192 | 240 | 244 | 137 | 141 | 144 | 152 | 0 | 0 | 179 | 179 |
| **CPg-ID045** | 154 | 154 | 185 | 185 | 192 | 200 | 268 | 280 | 251 | 255 | 298 | 334 | 230 | 230 | 156 | 156 | 0 | 0 | 240 | 0 | 137 | 141 | 144 | 152 | 158 | 170 | 179 | 179 |
| **CPg-ID046** | 142 | 154 | 173 | 193 | 192 | 200 | 268 | 280 | 251 | 271 | 298 | 334 | 230 | 242 | 152 | 152 | 176 | 196 | 244 | 244 | 137 | 145 | 148 | 152 | 0 | 0 | 179 | 183 |
| **CPg-ID047** | 146 | 150 | 193 | 193 | 196 | 200 | 280 | 280 | 267 | 271 | 322 | 322 | 242 | 242 | 148 | 156 | 196 | 196 | 244 | 244 | 141 | 141 | 144 | 148 | 170 | 178 | 179 | 191 |
| **CPg-ID048** | 146 | 154 | 181 | 193 | 192 | 196 | 260 | 260 | 259 | 0 | 326 | 334 | 0 | 0 | 152 | 152 | 0 | 0 | 0 | 0 | 137 | 145 | 140 | 140 | 166 | 178 | 0 | 0 |
| **CPg-ID055** | 150 | 150 | 181 | 185 | 192 | 196 | 268 | 280 | 251 | 259 | 318 | 326 | 242 | 242 | 152 | 156 | 0 | 0 | 0 | 0 | 133 | 145 | 144 | 144 | 0 | 0 | 171 | 171 |
| **CPg-ID056** | 146 | 146 | 181 | 193 | 192 | 196 | 280 | 280 | 251 | 259 | 314 | 326 | 230 | 242 | 148 | 156 | 176 | 184 | 244 | 248 | 137 | 141 | 144 | 144 | 174 | 174 | 183 | 191 |
| **CPg-ID057** | 142 | 154 | 185 | 193 | 192 | 0 | 268 | 268 | 251 | 271 | 0 | 0 | 0 | 0 | 152 | 156 | 172 | 172 | 244 | 244 | 137 | 137 | 0 | 0 | 0 | 0 | 179 | 183 |
| **CPg-ID058** | 142 | 150 | 193 | 193 | 192 | 196 | 272 | 280 | 251 | 251 | 334 | 334 | 230 | 274 | 152 | 152 | 176 | 196 | 240 | 244 | 141 | 145 | 140 | 148 | 166 | 166 | 179 | 183 |
| **CPg-ID059** | 154 | 154 | 173 | 185 | 192 | 200 | 264 | 268 | 251 | 271 | 298 | 334 | 242 | 274 | 152 | 156 | 192 | 196 | 244 | 244 | 137 | 137 | 144 | 152 | 158 | 170 | 179 | 183 |
| **CPg-ID060** | 150 | 150 | 173 | 185 | 192 | 196 | 268 | 276 | 255 | 275 | 314 | 330 | 242 | 246 | 152 | 156 | 0 | 0 | 0 | 0 | 137 | 145 | 144 | 148 | 0 | 0 | 179 | 183 |
| **CPg-ID061** | 146 | 154 | 185 | 193 | 196 | 200 | 268 | 280 | 271 | 271 | 314 | 334 | 234 | 238 | 148 | 152 | 192 | 192 | 0 | 0 | 137 | 141 | 144 | 144 | 162 | 166 | 179 | 179 |
| **CPg-ID062** | 154 | 162 | 193 | 193 | 176 | 196 | 0 | 0 | 251 | 251 | 322 | 334 | 234 | 234 | 136 | 136 | 168 | 168 | 0 | 0 | 129 | 145 | 0 | 0 | 174 | 174 | 0 | 0 |
| **CPg-ID063** | 146 | 154 | 185 | 189 | 192 | 196 | 264 | 272 | 255 | 279 | 314 | 318 | 230 | 242 | 140 | 148 | 180 | 180 | 236 | 244 | 137 | 141 | 144 | 148 | 162 | 162 | 171 | 179 |
| **CPg-ID064** | 146 | 146 | 185 | 193 | 192 | 200 | 264 | 280 | 251 | 271 | 314 | 318 | 242 | 246 | 140 | 152 | 184 | 188 | 244 | 248 | 137 | 137 | 144 | 144 | 162 | 162 | 183 | 187 |
| **CPg-ID065** | 146 | 146 | 185 | 193 | 196 | 196 | 268 | 280 | 247 | 275 | 322 | 334 | 234 | 274 | 148 | 152 | 176 | 188 | 232 | 248 | 141 | 145 | 144 | 144 | 166 | 166 | 183 | 183 |
| **CPg-ID066** | 150 | 150 | 185 | 185 | 200 | 200 | 264 | 280 | 247 | 271 | 334 | 334 | 238 | 274 | 140 | 156 | 192 | 192 | 244 | 244 | 137 | 141 | 144 | 144 | 158 | 162 | 191 | 191 |
| **CPg-ID067** | 150 | 154 | 181 | 189 | 180 | 196 | 260 | 272 | 267 | 267 | 322 | 334 | 0 | 0 | 148 | 152 | 180 | 184 | 244 | 244 | 141 | 145 | 140 | 140 | 170 | 178 | 183 | 183 |
| **CPg-ID068** | 154 | 154 | 185 | 189 | 200 | 200 | 0 | 280 | 251 | 267 | 334 | 334 | 230 | 274 | 152 | 156 | 184 | 188 | 240 | 244 | 137 | 141 | 140 | 144 | 158 | 162 | 179 | 183 |
| **CPg-ID069** | 154 | 154 | 185 | 185 | 196 | 200 | 268 | 268 | 271 | 275 | 318 | 330 | 238 | 242 | 152 | 156 | 0 | 0 | 244 | 244 | 137 | 137 | 140 | 144 | 158 | 166 | 179 | 191 |
| **CPg-ID070** | 146 | 146 | 173 | 189 | 192 | 196 | 276 | 280 | 259 | 271 | 306 | 314 | 230 | 238 | 148 | 148 | 188 | 200 | 240 | 244 | 141 | 153 | 140 | 152 | 166 | 178 | 183 | 183 |
| **CPg-ID071** | 146 | 154 | 173 | 177 | 192 | 200 | 260 | 280 | 271 | 275 | 298 | 314 | 230 | 234 | 148 | 160 | 188 | 188 | 244 | 244 | 141 | 145 | 140 | 152 | 166 | 178 | 179 | 183 |
| **CPg-ID074** | 146 | 158 | 177 | 181 | 196 | 200 | 264 | 268 | 251 | 271 | 334 | 334 | 242 | 274 | 148 | 152 | 176 | 196 | 244 | 248 | 137 | 137 | 140 | 148 | 162 | 166 | 183 | 187 |
| **CPg-ID077** | 146 | 154 | 181 | 185 | 188 | 192 | 268 | 280 | 271 | 275 | 306 | 314 | 242 | 242 | 140 | 156 | 176 | 176 | 244 | 244 | 141 | 141 | 140 | 148 | 166 | 166 | 179 | 187 |
| **CPg-ID081** | 154 | 154 | 193 | 193 | 196 | 200 | 280 | 280 | 247 | 271 | 314 | 314 | 234 | 274 | 148 | 156 | 188 | 192 | 248 | 248 | 137 | 137 | 144 | 144 | 162 | 166 | 183 | 183 |
| **CPg-ID084** | 154 | 154 | 189 | 193 | 200 | 200 | 254 | 280 | 251 | 267 | 322 | 322 | 234 | 274 | 152 | 152 | 184 | 188 | 244 | 248 | 137 | 137 | 144 | 144 | 162 | 162 | 183 | 183 |
| **CPg-ID086** | 154 | 154 | 185 | 185 | 200 | 200 | 254 | 0 | 251 | 263 | 334 | 334 | 230 | 274 | 146 | 0 | 0 | 0 | 230 | 248 | 141 | 141 | 140 | 144 | 162 | 166 | 183 | 183 |
| **CPg-ID088** | 150 | 154 | 173 | 185 | 200 | 200 | 264 | 268 | 263 | 0 | 322 | 334 | 0 | 0 | 0 | 0 | 0 | 0 | 0 | 0 | 137 | 137 | 0 | 0 | 0 | 0 | 183 | 183 |
| **CPg-ID089** | 150 | 154 | 185 | 185 | 200 | 204 | 268 | 280 | 255 | 267 | 306 | 334 | 234 | 242 | 152 | 152 | 184 | 192 | 240 | 248 | 133 | 137 | 144 | 148 | 162 | 166 | 183 | 187 |
| **CPg-ID090** | 150 | 150 | 185 | 197 | 180 | 200 | 268 | 268 | 263 | 271 | 298 | 334 | 230 | 230 | 156 | 156 | 0 | 0 | 0 | 0 | 137 | 141 | 0 | 0 | 0 | 0 | 0 | 0 |
| **CPg-ID091** | 146 | 150 | 177 | 181 | 196 | 200 | 272 | 280 | 251 | 263 | 314 | 318 | 234 | 234 | 152 | 156 | 180 | 196 | 244 | 244 | 141 | 141 | 144 | 144 | 0 | 0 | 183 | 187 |
| **CPg-ID092** | 146 | 154 | 193 | 193 | 192 | 196 | 280 | 284 | 251 | 271 | 298 | 298 | 226 | 242 | 148 | 156 | 192 | 196 | 248 | 248 | 137 | 137 | 140 | 144 | 166 | 166 | 183 | 183 |
| **CPg-ID093** | 146 | 150 | 193 | 193 | 180 | 196 | 280 | 280 | 251 | 271 | 322 | 322 | 242 | 242 | 148 | 0 | 196 | 196 | 232 | 244 | 141 | 141 | 144 | 144 | 0 | 0 | 179 | 191 |
| **CPg-ID094** | 146 | 146 | 185 | 193 | 192 | 200 | 280 | 280 | 267 | 267 | 318 | 330 | 230 | 242 | 152 | 156 | 188 | 188 | 244 | 248 | 141 | 145 | 144 | 144 | 166 | 170 | 179 | 191 |
| **CPg-ID097** | 146 | 154 | 181 | 193 | 196 | 196 | 268 | 280 | 259 | 271 | 334 | 334 | 234 | 274 | 148 | 148 | 176 | 180 | 244 | 252 | 137 | 145 | 148 | 148 | 166 | 166 | 179 | 187 |
| **CPg-ID100** | 146 | 146 | 181 | 181 | 196 | 196 | 270 | 278 | 259 | 271 | 314 | 326 | 234 | 242 | 148 | 156 | 180 | 188 | 244 | 244 | 137 | 141 | 144 | 144 | 166 | 178 | 171 | 191 |
| **CPg-ID101** | 154 | 154 | 185 | 193 | 192 | 200 | 272 | 280 | 251 | 271 | 314 | 334 | 230 | 238 | 144 | 152 | 180 | 192 | 244 | 252 | 137 | 137 | 144 | 144 | 162 | 166 | 179 | 183 |
| **CPg-ID102** | 150 | 154 | 185 | 193 | 192 | 192 | 272 | 280 | 251 | 251 | 334 | 334 | 230 | 234 | 144 | 152 | 180 | 196 | 240 | 244 | 137 | 145 | 144 | 144 | 166 | 166 | 183 | 183 |
| **CPg-ID103** | 146 | 154 | 181 | 185 | 192 | 196 | 280 | 280 | 251 | 271 | 334 | 334 | 234 | 238 | 148 | 152 | 180 | 196 | 244 | 252 | 141 | 145 | 144 | 144 | 166 | 166 | 171 | 183 |
| **CPg-ID104** | 154 | 154 | 181 | 185 | 196 | 200 | 262 | 280 | 251 | 271 | 314 | 334 | 238 | 242 | 152 | 156 | 192 | 192 | 244 | 244 | 137 | 141 | 140 | 148 | 162 | 166 | 171 | 183 |
| **CPg-ID107** | 146 | 146 | 181 | 193 | 180 | 200 | 276 | 280 | 271 | 271 | 298 | 334 | 234 | 234 | 148 | 152 | 172 | 188 | 232 | 248 | 137 | 145 | 140 | 144 | 162 | 166 | 171 | 183 |
| **CPg-ID108** | 150 | 158 | 185 | 193 | 196 | 200 | 272 | 280 | 267 | 271 | 306 | 334 | 234 | 242 | 148 | 152 | 192 | 192 | 240 | 252 | 137 | 137 | 144 | 148 | 166 | 166 | 179 | 183 |
| **CPg-ID113** | 154 | 158 | 0 | 185 | 169 | 200 | 0 | 0 | 251 | 251 | 298 | 334 | 230 | 230 | 0 | 0 | 0 | 0 | 0 | 0 | 0 | 0 | 0 | 0 | 0 | 0 | 0 | 0 |
| **CPg-ID114** | 146 | 146 | 0 | 193 | 0 | 0 | 270 | 280 | 0 | 273 | 330 | 330 | 242 | 242 | 0 | 0 | 0 | 0 | 0 | 0 | 137 | 141 | 144 | 144 | 0 | 0 | 179 | 191 |
| **CPg-ID115** | 146 | 154 | 185 | 189 | 192 | 200 | 258 | 270 | 255 | 271 | 298 | 330 | 242 | 274 | 152 | 156 | 196 | 196 | 232 | 244 | 137 | 137 | 144 | 144 | 158 | 166 | 179 | 179 |
| **CPg-ID116** | 150 | 154 | 177 | 193 | 196 | 200 | 266 | 280 | 271 | 271 | 298 | 318 | 242 | 242 | 144 | 152 | 188 | 188 | 232 | 0 | 137 | 145 | 144 | 144 | 162 | 166 | 179 | 187 |
| **CPg-ID117** | 146 | 146 | 193 | 193 | 0 | 0 | 280 | 280 | 247 | 247 | 0 | 0 | 234 | 274 | 148 | 152 | 176 | 188 | 232 | 232 | 141 | 145 | 144 | 144 | 0 | 0 | 183 | 183 |
| **CPg-ID118** | 154 | 158 | 177 | 177 | 192 | 200 | 266 | 270 | 251 | 271 | 298 | 334 | 242 | 242 | 156 | 156 | 176 | 192 | 244 | 244 | 137 | 137 | 148 | 152 | 170 | 170 | 183 | 195 |
| **CPg-ID119** | 146 | 154 | 185 | 193 | 180 | 196 | 262 | 280 | 251 | 255 | 334 | 334 | 234 | 234 | 144 | 152 | 176 | 188 | 232 | 244 | 137 | 137 | 140 | 144 | 166 | 166 | 183 | 183 |
| **CPg-ID120** | 146 | 150 | 181 | 185 | 200 | 200 | 272 | 276 | 275 | 275 | 318 | 326 | 242 | 242 | 140 | 148 | 176 | 176 | 240 | 240 | 137 | 137 | 140 | 144 | 162 | 166 | 179 | 183 |
| **CPg-ID121** | 146 | 158 | 189 | 193 | 196 | 200 | 266 | 280 | 271 | 271 | 298 | 334 | 238 | 242 | 152 | 152 | 184 | 188 | 232 | 240 | 137 | 141 | 144 | 144 | 0 | 0 | 179 | 183 |
| **CPg-ID122** | 146 | 154 | 189 | 193 | 196 | 196 | 0 | 288 | 255 | 259 | 326 | 334 | 270 | 274 | 0 | 0 | 0 | 0 | 0 | 0 | 137 | 141 | 0 | 0 | 0 | 0 | 0 | 0 |
| **CPg-ID123** | 154 | 154 | 181 | 193 | 196 | 196 | 272 | 280 | 263 | 267 | 314 | 334 | 274 | 274 | 144 | 152 | 172 | 196 | 240 | 240 | 137 | 137 | 140 | 148 | 178 | 178 | 179 | 179 |
| **CPg-ID126** | 146 | 146 | 177 | 193 | 192 | 200 | 276 | 280 | 251 | 271 | 314 | 334 | 234 | 274 | 148 | 152 | 172 | 188 | 244 | 248 | 137 | 137 | 144 | 144 | 162 | 166 | 171 | 183 |
| **CPg-ID127** | 150 | 158 | 177 | 193 | 200 | 200 | 266 | 280 | 271 | 275 | 298 | 302 | 242 | 274 | 152 | 156 | 0 | 0 | 240 | 244 | 137 | 137 | 144 | 148 | 0 | 0 | 179 | 195 |
| **CPg-ID128** | 146 | 146 | 185 | 193 | 200 | 200 | 270 | 280 | 267 | 271 | 330 | 330 | 242 | 242 | 152 | 156 | 188 | 196 | 232 | 244 | 137 | 141 | 144 | 144 | 158 | 166 | 179 | 191 |
| **CPg-ID129** | 146 | 146 | 181 | 189 | 192 | 192 | 266 | 276 | 271 | 279 | 314 | 318 | 230 | 242 | 148 | 156 | 176 | 180 | 236 | 244 | 137 | 141 | 144 | 148 | 166 | 166 | 179 | 187 |
| **CPg-ID130** | 146 | 154 | 181 | 181 | 192 | 196 | 266 | 274 | 267 | 271 | 318 | 322 | 242 | 242 | 148 | 148 | 0 | 0 | 0 | 0 | 137 | 141 | 140 | 144 | 166 | 166 | 179 | 183 |
| **CPg-ID131** | 146 | 146 | 189 | 193 | 192 | 196 | 276 | 284 | 251 | 271 | 314 | 326 | 230 | 274 | 156 | 156 | 0 | 0 | 236 | 248 | 137 | 141 | 144 | 148 | 0 | 0 | 183 | 187 |
| **CPg-ID132** | 146 | 154 | 189 | 189 | 192 | 200 | 266 | 274 | 271 | 279 | 318 | 326 | 230 | 234 | 148 | 160 | 180 | 180 | 236 | 244 | 137 | 141 | 144 | 148 | 166 | 166 | 187 | 191 |
| **CPg-ID134** | 146 | 154 | 193 | 193 | 180 | 192 | 280 | 280 | 255 | 267 | 298 | 330 | 230 | 234 | 152 | 152 | 188 | 196 | 232 | 248 | 137 | 145 | 140 | 144 | 166 | 166 | 179 | 183 |
| **CPg-ID135** | 154 | 154 | 181 | 193 | 196 | 200 | 266 | 280 | 259 | 267 | 330 | 334 | 234 | 274 | 148 | 152 | 176 | 196 | 0 | 0 | 137 | 145 | 148 | 148 | 170 | 178 | 187 | 195 |
| **CPg-ID136** | 146 | 146 | 181 | 185 | 200 | 200 | 276 | 280 | 267 | 275 | 322 | 326 | 242 | 242 | 148 | 156 | 176 | 180 | 240 | 260 | 137 | 137 | 140 | 148 | 166 | 170 | 179 | 183 |
| **CPg-ID137** | 146 | 146 | 181 | 193 | 200 | 204 | 266 | 284 | 263 | 267 | 318 | 318 | 230 | 234 | 156 | 160 | 176 | 0 | 236 | 244 | 137 | 145 | 144 | 144 | 158 | 166 | 195 | 195 |
| **CPg-ID138** | 146 | 146 | 193 | 193 | 196 | 200 | 280 | 284 | 267 | 267 | 314 | 314 | 230 | 238 | 152 | 152 | 176 | 184 | 240 | 244 | 137 | 137 | 144 | 144 | 170 | 170 | 183 | 195 |
| **CPg-ID139** | 146 | 146 | 189 | 193 | 192 | 200 | 262 | 270 | 251 | 255 | 322 | 334 | 230 | 230 | 148 | 152 | 188 | 192 | 248 | 248 | 137 | 145 | 140 | 144 | 170 | 170 | 183 | 187 |
| **CPg-ID140** | 150 | 154 | 185 | 185 | 196 | 200 | 268 | 280 | 247 | 275 | 330 | 334 | 242 | 274 | 152 | 156 | 176 | 192 | 244 | 244 | 137 | 141 | 140 | 144 | 158 | 166 | 183 | 191 |
| **CPg-ID141** | 146 | 154 | 181 | 193 | 200 | 200 | 266 | 276 | 255 | 267 | 298 | 318 | 234 | 234 | 160 | 160 | 184 | 188 | 236 | 244 | 137 | 145 | 144 | 144 | 166 | 170 | 179 | 195 |
| **CPg-ID142** | 146 | 154 | 181 | 193 | 196 | 196 | 264 | 280 | 251 | 263 | 306 | 326 | 246 | 274 | 148 | 148 | 184 | 184 | 244 | 256 | 137 | 141 | 144 | 148 | 166 | 174 | 179 | 187 |
| **CPg-ID143** | 146 | 146 | 177 | 181 | 196 | 200 | 276 | 280 | 255 | 279 | 298 | 298 | 234 | 234 | 152 | 160 | 184 | 188 | 244 | 248 | 137 | 137 | 152 | 156 | 170 | 178 | 179 | 179 |
| **CPg-ID144** | 146 | 146 | 181 | 193 | 192 | 192 | 270 | 274 | 251 | 259 | 318 | 322 | 242 | 242 | 148 | 152 | 192 | 192 | 244 | 248 | 137 | 145 | 144 | 144 | 166 | 166 | 171 | 183 |
| **CPg-ID145** | 146 | 150 | 177 | 193 | 200 | 200 | 276 | 276 | 267 | 271 | 322 | 326 | 234 | 234 | 148 | 156 | 180 | 184 | 232 | 244 | 137 | 141 | 148 | 148 | 170 | 174 | 179 | 179 |
| **CPg-ID146** | 146 | 154 | 181 | 193 | 192 | 196 | 270 | 274 | 251 | 267 | 298 | 334 | 234 | 274 | 140 | 152 | 0 | 0 | 240 | 240 | 137 | 145 | 140 | 148 | 158 | 166 | 183 | 183 |
| **CPg-ID147** | 146 | 146 | 181 | 181 | 192 | 192 | 256 | 270 | 275 | 275 | 326 | 330 | 242 | 242 | 152 | 152 | 0 | 0 | 0 | 0 | 141 | 145 | 0 | 0 | 0 | 0 | 179 | 179 |
| **CPg-ID148** | 146 | 154 | 189 | 193 | 192 | 200 | 266 | 280 | 267 | 267 | 298 | 314 | 230 | 242 | 156 | 156 | 172 | 0 | 240 | 244 | 141 | 145 | 140 | 144 | 158 | 166 | 183 | 191 |
| **CPg-ID149** | 150 | 154 | 177 | 181 | 180 | 196 | 272 | 276 | 259 | 267 | 318 | 322 | 230 | 242 | 152 | 152 | 0 | 0 | 0 | 0 | 137 | 141 | 140 | 144 | 166 | 170 | 183 | 187 |
| **CPg-ID150** | 146 | 154 | 181 | 193 | 192 | 196 | 268 | 276 | 251 | 267 | 298 | 306 | 246 | 274 | 148 | 156 | 184 | 196 | 244 | 244 | 137 | 137 | 140 | 148 | 166 | 166 | 183 | 187 |
| **CPg-ID151** | 150 | 150 | 185 | 185 | 200 | 200 | 276 | 280 | 251 | 271 | 314 | 326 | 246 | 274 | 152 | 152 | 176 | 196 | 244 | 248 | 145 | 145 | 140 | 148 | 162 | 166 | 179 | 187 |
| **CPg-ID152** | 146 | 150 | 185 | 193 | 200 | 200 | 280 | 280 | 267 | 267 | 318 | 322 | 230 | 242 | 156 | 156 | 0 | 0 | 0 | 0 | 141 | 145 | 144 | 144 | 170 | 170 | 179 | 191 |
| **CPg-ID153** | 150 | 154 | 177 | 189 | 196 | 200 | 270 | 280 | 255 | 271 | 298 | 322 | 234 | 242 | 144 | 148 | 0 | 0 | 244 | 0 | 137 | 145 | 140 | 148 | 162 | 166 | 183 | 187 |
| **CPg-ID154** | 150 | 154 | 185 | 189 | 192 | 196 | 264 | 272 | 255 | 263 | 314 | 318 | 242 | 242 | 140 | 152 | 180 | 184 | 240 | 244 | 137 | 141 | 144 | 144 | 150 | 162 | 171 | 179 |
| **CPg-ID155** | 150 | 158 | 185 | 193 | 192 | 200 | 270 | 280 | 251 | 259 | 334 | 334 | 242 | 246 | 140 | 140 | 0 | 0 | 244 | 244 | 145 | 145 | 140 | 144 | 162 | 170 | 183 | 187 |
| **CPg-ID156** | 146 | 154 | 177 | 193 | 196 | 196 | 280 | 280 | 267 | 271 | 318 | 334 | 230 | 274 | 148 | 152 | 0 | 0 | 240 | 244 | 137 | 141 | 144 | 148 | 166 | 166 | 179 | 195 |
| **CPg-ID157** | 154 | 154 | 177 | 189 | 200 | 200 | 0 | 0 | 255 | 271 | 298 | 298 | 234 | 0 | 0 | 0 | 0 | 0 | 0 | 0 | 137 | 145 | 140 | 0 | 0 | 166 | 179 | 183 |
| **CPg-ID158** | 146 | 154 | 185 | 193 | 192 | 196 | 266 | 280 | 267 | 267 | 298 | 318 | 242 | 242 | 152 | 152 | 172 | 184 | 244 | 248 | 141 | 145 | 144 | 144 | 166 | 166 | 179 | 187 |
